# Inhibition of the extracellular enzyme A disintegrin and metalloprotease with thrombospondin motif 4 prevents cardiac fibrosis and dysfunction

**DOI:** 10.1093/cvr/cvad078

**Published:** 2023-05-22

**Authors:** Maria Vistnes, Pugazendhi Murugan Erusappan, Athiramol Sasi, Einar Sjaastad Nordén, Kaja Knudsen Bergo, Andreas Romaine, Ida Gjervold Lunde, Lili Zhang, Maria Belland Olsen, Jonas Øgaard, Cathrine Rein Carlson, Christian Hjorth Wang, Jon Riise, Christen Peder Dahl, Arnt Eltvedt Fiane, Ida Marie Hauge-Iversen, Emil Espe, Arne Olav Melleby, Theis Tønnessen, Jan Magnus Aronsen, Ivar Sjaastad, Geir Christensen

**Affiliations:** Institute for Experimental Medical Research, Oslo University Hospital and University of Oslo, Kirkeveien 166, 0450 Oslo, Norway; K.G. Jebsen Center for Cardiac Research, University of Oslo, Kirkeveien 166, 0450 Oslo, Norway; Department of Cardiology, Oslo University Hospital Ullevål, Kirkeveien 166, 0450 Oslo, Norway; Department of Internal Medicine, Diakonhjemmet Hospital, Diakonveien 12, 0370 Oslo, Norway; Institute for Experimental Medical Research, Oslo University Hospital and University of Oslo, Kirkeveien 166, 0450 Oslo, Norway; K.G. Jebsen Center for Cardiac Research, University of Oslo, Kirkeveien 166, 0450 Oslo, Norway; Institute for Experimental Medical Research, Oslo University Hospital and University of Oslo, Kirkeveien 166, 0450 Oslo, Norway; K.G. Jebsen Center for Cardiac Research, University of Oslo, Kirkeveien 166, 0450 Oslo, Norway; Institute for Experimental Medical Research, Oslo University Hospital and University of Oslo, Kirkeveien 166, 0450 Oslo, Norway; K.G. Jebsen Center for Cardiac Research, University of Oslo, Kirkeveien 166, 0450 Oslo, Norway; Institute for Experimental Medical Research, Oslo University Hospital and University of Oslo, Kirkeveien 166, 0450 Oslo, Norway; K.G. Jebsen Center for Cardiac Research, University of Oslo, Kirkeveien 166, 0450 Oslo, Norway; Institute for Experimental Medical Research, Oslo University Hospital and University of Oslo, Kirkeveien 166, 0450 Oslo, Norway; K.G. Jebsen Center for Cardiac Research, University of Oslo, Kirkeveien 166, 0450 Oslo, Norway; Institute for Experimental Medical Research, Oslo University Hospital and University of Oslo, Kirkeveien 166, 0450 Oslo, Norway; K.G. Jebsen Center for Cardiac Research, University of Oslo, Kirkeveien 166, 0450 Oslo, Norway; Institute for Experimental Medical Research, Oslo University Hospital and University of Oslo, Kirkeveien 166, 0450 Oslo, Norway; K.G. Jebsen Center for Cardiac Research, University of Oslo, Kirkeveien 166, 0450 Oslo, Norway; Research Institute of Internal Medicine, Oslo University Hospital and University of Oslo, Sognsvannsveien 20, 0372 Oslo, Norway; Research Institute of Internal Medicine, Oslo University Hospital and University of Oslo, Sognsvannsveien 20, 0372 Oslo, Norway; Institute for Experimental Medical Research, Oslo University Hospital and University of Oslo, Kirkeveien 166, 0450 Oslo, Norway; Department of Internal Medicine, Diakonhjemmet Hospital, Diakonveien 12, 0370 Oslo, Norway; Department of Oncology, Oslo University Hospital, Ullernchausseen 70, 0379 Oslo, Norway; Department of Cardiology, Oslo University Hospital Rikshospitalet, Sognsvannsveien 20, 0372 Oslo, Norway; Department of Cardiothoracic Surgery, Oslo University Hospital, Sognsvannsveien 20, 0372 Oslo, Norway; Faculty of Medicine, University of Oslo, Klaus Torgårdsvei 3, 0372 Oslo, Norway; Institute for Experimental Medical Research, Oslo University Hospital and University of Oslo, Kirkeveien 166, 0450 Oslo, Norway; K.G. Jebsen Center for Cardiac Research, University of Oslo, Kirkeveien 166, 0450 Oslo, Norway; Institute for Experimental Medical Research, Oslo University Hospital and University of Oslo, Kirkeveien 166, 0450 Oslo, Norway; K.G. Jebsen Center for Cardiac Research, University of Oslo, Kirkeveien 166, 0450 Oslo, Norway; Institute for Experimental Medical Research, Oslo University Hospital and University of Oslo, Kirkeveien 166, 0450 Oslo, Norway; Department of Molecular Medicine, Institute of Basic Medical Sciences, University of Oslo, Sognsvannsveien 9, 0372 Oslo, Norway; Institute for Experimental Medical Research, Oslo University Hospital and University of Oslo, Kirkeveien 166, 0450 Oslo, Norway; K.G. Jebsen Center for Cardiac Research, University of Oslo, Kirkeveien 166, 0450 Oslo, Norway; Department of Cardiothoracic Surgery, Oslo University Hospital, Sognsvannsveien 20, 0372 Oslo, Norway; Faculty of Medicine, University of Oslo, Klaus Torgårdsvei 3, 0372 Oslo, Norway; Department of Molecular Medicine, Institute of Basic Medical Sciences, University of Oslo, Sognsvannsveien 9, 0372 Oslo, Norway; Department of Pharmacology, Oslo University Hospital Rikshospitalet, Sognsvannsveien 20, 0372 Oslo, Norway; Institute for Experimental Medical Research, Oslo University Hospital and University of Oslo, Kirkeveien 166, 0450 Oslo, Norway; K.G. Jebsen Center for Cardiac Research, University of Oslo, Kirkeveien 166, 0450 Oslo, Norway; Institute for Experimental Medical Research, Oslo University Hospital and University of Oslo, Kirkeveien 166, 0450 Oslo, Norway; K.G. Jebsen Center for Cardiac Research, University of Oslo, Kirkeveien 166, 0450 Oslo, Norway

**Keywords:** New therapy, Cardiac fibrosis, Extracellular matrix, Heart failure, ADAMTS enzymes

## Abstract

**Aims:**

Heart failure is a condition with high mortality rates, and there is a lack of therapies that directly target maladaptive changes in the extracellular matrix (ECM), such as fibrosis. We investigated whether the ECM enzyme known as *A disintegrin and metalloprotease with thrombospondin motif* (ADAMTS) 4 might serve as a therapeutic target in treatment of heart failure and cardiac fibrosis.

**Methods and results:**

The effects of pharmacological ADAMTS4 inhibition on cardiac function and fibrosis were examined in rats exposed to cardiac pressure overload. Disease mechanisms affected by the treatment were identified based on changes in the myocardial transcriptome. Following aortic banding, rats receiving an ADAMTS inhibitor, with high inhibitory capacity for ADAMTS4, showed substantially better cardiac function than vehicle-treated rats, including ∼30% reduction in E/e′ and left atrial diameter, indicating an improvement in diastolic function. ADAMTS inhibition also resulted in a marked reduction in myocardial collagen content and a down-regulation of transforming growth factor (TGF)-β target genes. The mechanism for the beneficial effects of ADAMTS inhibition was further studied in cultured human cardiac fibroblasts producing mature ECM. ADAMTS4 caused a 50% increase in the TGF-β levels in the medium. Simultaneously, ADAMTS4 elicited a not previously known cleavage of TGF-β-binding proteins, i.e. latent-binding protein of TGF-β and extra domain A-fibronectin. These effects were abolished by the ADAMTS inhibitor. In failing human hearts, we observed a marked increase in ADAMTS4 expression and cleavage activity.

**Conclusion:**

Inhibition of ADAMTS4 improves cardiac function and reduces collagen accumulation in rats with cardiac pressure overload, possibly through a not previously known cleavage of molecules that control TGF-β availability. Targeting ADAMTS4 may serve as a novel strategy in heart failure treatment, in particular, in heart failure with fibrosis and diastolic dysfunction.

## Graphical Abstract

**Graphical Abstract:**
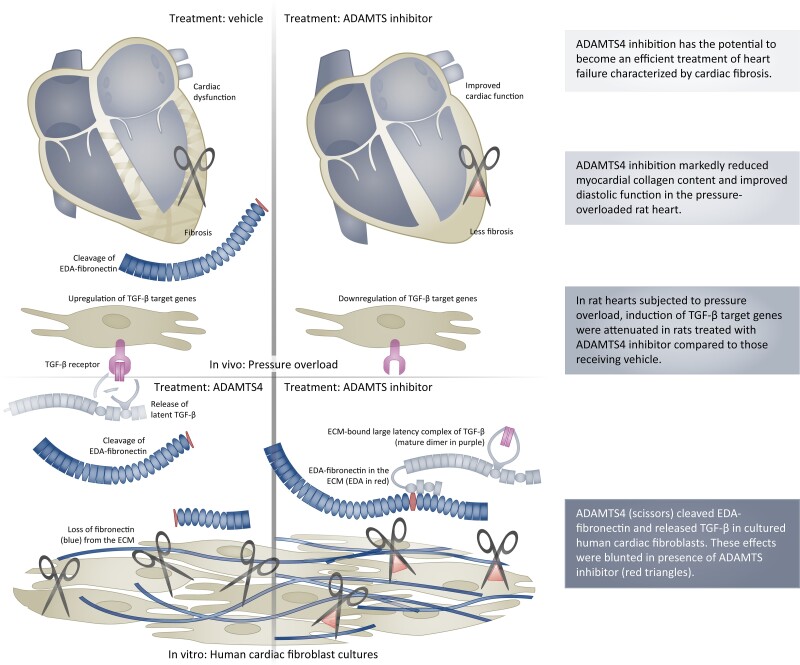
ADAMTS4 inhibition prevents cardiac fibrosis by reducing the release of ECM-bound TGF-β due to EDA-fibronectin cleavage. TGF, transforming growth factor; ADAMTS4, a disintegrin and metalloprotease with thrombospondin motif; EDA, extra domain A; ECM, extracellular matrix.

## 1. Introduction

Heart failure is a major public health problem due to its increasing prevalence and high mortality rates.^1–3^ Maladaptive remodelling and the excessive deposition of the extracellular matrix (ECM) seen in fibrosis are common responses to increased afterload, as observed in hypertension or aortic stenosis. Cardiac fibrosis attenuates cardiac function and accelerates the development of heart failure and is associated with poor outcomes.^4–6^ The unmet need for therapies that target the cardiac ECM has been highlighted by the scientific community^4,7,8^ and leading medical societies.^9,10^

Maladaptive remodelling of the ECM is driven by altered proteolysis and synthesis of its components.^11^ Secreted metalloproteases such as *A disintegrin and metalloprotease with thrombospondin motif* (ADAMTS) are potential contributors to cardiac ECM remodelling via their preference for cleaving glycosylated proteins.^12^ ADAMTS4 is expressed by fibroblasts in the healthy human^13^ and failing murine heart^14^ and is up-regulated in the fibrotic injured heart^15^ and cardiac cells in response to inflammatory stimuli.^16^ We have previously demonstrated beneficial effects of reducing ADAMTS-mediated versicanase activity in the pressure-overloaded heart.^16^ However, the effects of more selective targeting of ADAMTS enzymes, and the mechanism for a potential beneficial effect of ADAMTS4 inhibition in cardiac disease, have not been explored.

We hypothesized that ADAMTS4 is a therapeutic target in cardiac dysfunction and fibrosis, due to its effects on ECM components. In the present study, we have used a small molecule that inhibits ADAMTS enzymes with a high potency for ADAMTS4 inhibition^17^ to determine the therapeutic potential and main molecular effects of ADAMTS4 inhibition in pressure-overloaded rat hearts. We demonstrate that pressure-overloaded rat hearts treated with the ADAMTS inhibitor are protected against fibrosis and cardiac dysfunction, and we reveal a novel role for ADAMTS4 in the mobilisation of latent transforming growth factor (TGF)-β through cleavage of extracellular domain A (EDA)-fibronectin. The translational potential of ADAMTS4 as a therapeutic target is supported by the findings of the beneficial effects of ADAMTS4 inhibition in rat hearts following pressure overload and that substantially increased ADAMTS4 activity is observed in human heart failure.

## 2. Methods

### 2.1 *In vivo* study design

The effect of the ADAMTS inhibitor on cardiac function in the pressure-overloaded heart was examined in rats that were subjected to aortic banding (AB) or to sham operation. In total, 24 animals were included in each AB group, whereas six rats were included in each sham group. To ensure an even distribution of the degree of stenosis in the AB groups, the rats were stratified and randomized to the treatments according to echocardiographic measures that were taken on Day 1 after surgery (see Supplementary material*online, Table S1*).

### 2.2 AB rat model

For the ADAMTS inhibitor treatment study, male Wistar rats (∼7–8 weeks of age, Janvier Labs, France) underwent AB through placement of a suture around the ascending aorta, as previously described.^18^ For ADAMTS expression analyses, we employed male Sprague Dawley rats (∼4–5 weeks of age, Janvier Labs, France) that underwent AB using an o-ring around the ascending aorta, as described.^19^ Sham rats that received a loose suture around the aorta (ADAMTS inhibitor treatment study) or received surgery without the insertion of o-ring (rats for expression analyses) served as controls. During surgery, rats were intubated and fully sedated by ventilation with a mixture of 98% oxygen and 2% isoflurane on a Zoovent (Triumph Technical Services, Milton Keynes, UK). Subcutaneous administration of 0.1 mg/kg buprenorphine was given as pre- and post-operative analgesia. All experiments were performed in accordance with the Norwegian Animal Welfare Act and the National Institutes of Health guidelines^20^ that conform to European Parliament Directive,^21^ and after approval from the Norwegian Animal Research Authority (FOTS ID 7737 and FOTS ID 20208).

### 2.3 Treatment with ADAMTS inhibitor

The hydroxamate-based small molecule designated ‘13n’, here called ‘ADAMTS inhibitor’, was generously provided free of charge by AstraZeneca (see *Conflict of interest*).^17^ Pharmacokinetic studies and simulations demonstrated that a dose of 15 mg/kg/day of the ADAMTS inhibitor yielded an unbound plasma concentration in the steady state of 270 nM (see Supplementary material*online, Figure S1* and *Table S2*), anticipated to lead to a similar organ level.^22^ Based on a previously reported IC_50_ of 26 nM for ADAMTS4 and 860 nM for ADAMTS5,^17^ the estimated plasma concentration was 10 times the reported half-maximal inhibitory concentration (IC_50_) for ADAMTS4, which indicated sufficient efficacy in the case of ADAMTS4, while constituting 30% of the IC_50_ for ADAMTS5 and 1% of the IC_50_ for matrix metalloproteinase-2.^17^ A microsuspension of the ADAMTS inhibitor or vehicle was administered by oral gavage once daily starting from Day 3 post-AB.

### 2.4 Echocardiography, magnetic resonance imaging and tissue harvest

We performed echocardiography and magnetic resonance imaging (MRI) 8 weeks after surgery to assess cardiac structure and function, as has been described previously.^23^ In brief, the rats underwent MRI examination on a 9.4T MR system (Agilent Technologies, Inc., USA) under anaesthesia with 1.5–2% isoflurane. Body temperature was kept around 37°C by warm air. Cine-MRI was acquired in the true short-axis and the four-chamber long-axis orientation in an electrocardiogram and respiratory gated manner. The rats were anaesthetized with 5% isoflurane before being euthanized by cardiac exsanguination. After the left ventricle and septum had been separated from the right ventricle, the cardiac ventricles and lungs were weighed and stored for later analyses.

### 2.5 Fibrosis, protein and mRNA quantification in AB rats

Collagen content was determined by the use of high performance liquid chromatography (HPLC), while the amounts of fibronectin, latent TGF-β-binding protein (LTBP) 1, and versican DPEAAE-immunoreactive fragments were determined by immunoblotting of ECM-rich fractions. The RNA was isolated and analysed by RT–qPCR to determine the expression of genes that encode ADAMTS enzymes that cleave proteoglycans, and genes associated with heart failure mechanisms. For the RNA sequencing, we used pooled samples taken from three representative rats in each AB group. A Benjamini–Hochberg false discovery rate (*q*-values) <0.15 was used to define the transcripts as differentially expressed genes (DEGs). The list of DEGs was annotated through the use of gene ontology (GO) enrichment analysis using the statistical overrepresentation test in g:Profiler (https://biit.cs.ut.ee/gprofiler/gost) and analysed through the use of Ingenuity pathways analysis (IPA) (Qiagen, MD) to identify potential upstream regulators.

### 2.6 Cultures of cardiac fibroblasts

Primary foetal and adult human cardiac fibroblasts were cultured for 7 days, as has been previously described,^24^ before treatment with 10 nM recombinant ADAMTS4 alone or in combination with 26 nM ADAMTS inhibitor. Due to a higher proliferation rate and less activated phenotype, foetal cells were employed for assessing the effects of ADAMTS4 or the ADAMTS inhibitor, except for the transfected mink lung cells (tMLC) assay where a higher level of latent TGF-β in the ECM was wanted. For siRNA experiments, primary foetal human cardiac fibroblasts were treated with non-targeting control siRNA or ADAMTS4 siRNA (s18228, Silencer® Select, Invitrogen) at a concentration of 10 nM according to manufacturer’s instructions and cultured for 7 days. Conditioned cell culture medium and cellular lysates were collected for protein detection by immunoblotting and immunohistochemistry, or for quantification of TGF-β by use of tMLC, as described previously.^25^ Adult human cardiac fibroblasts were also exposed to mechanical stress of 10% biaxial strain at a frequency of 1 Hz for 24 h.

### 2.7 Human hearts

Biobanked human myocardial samples were used to determine levels of ADAMTS4 expression and activity. Samples were taken from the free wall of the left ventricle of explanted hearts from patients with dilated non-ischaemic cardiomyopathy and from donor hearts that had not been used for transplantation due to no Scandinavian recipients being available. Tissue lysates were first solubilized using the compartment protein extraction kit (2145, Millipore, MA) according to the manufacturer’s instructions. ECM fractions were extracted as previously described^26^ and reported in *Supplementary Methods*. The use of the samples was approved by the regional ethics committee (REK 2014/569 and 07482a).

### 2.8 Statistical analyses

For comparisons between groups in the rat study, we employed one-way analysis of variance (ANOVA) with planned comparisons to detect differences between the following groups: sham vehicle vs. sham ADAMTS inhibitor, sham vehicle vs. AB vehicle, and AB vehicle vs. AB ADAMTS inhibitor. Bonferroni correction for these three comparisons was performed, and results were regarded as significant for *P* < 0.05. For cell culture experiments, comparisons were performed via ANOVA for correlated samples, with Bonferroni corrections as described above. For analyses in cellular lysates and immunohistochemistry, one-way ANOVA with planned comparisons and Bonferroni *post hoc* corrections were performed, whereas Student’s *t*-tests were applied in human samples for comparisons between two groups. Log-transformation was performed to achieve equal variances (<3 standard deviation (SD) difference between groups) and/or normal distribution, as appropriate. Data are presented as mean ± 1 SD. Survival of AB rats was assessed by Cox regression and log-rank test. All statistical analyses were performed in SPSS 27 (IBM, NY), graphs were prepared in GraphPad Prism 9 (GraphPad Software, CA), and figure panels were assembled and illustrations prepared in Adobe Illustrator 16.0 (Adobe Systems, CA).

## 3. Results

### 3.1 ADAMTS inhibition prevented cardiac dysfunction in pressure-overloaded rat hearts

**Figure 1:**
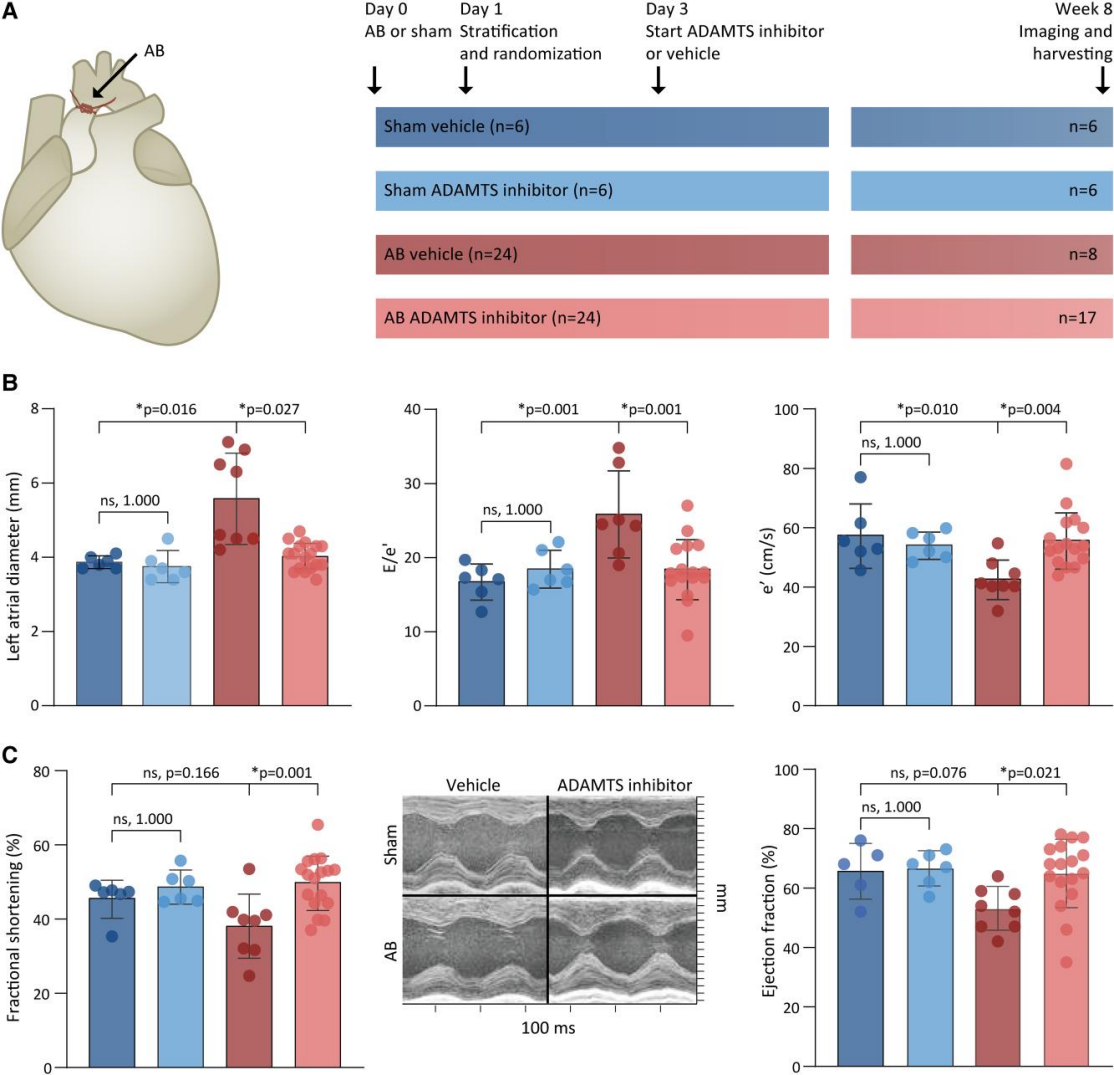
Study design and effects of ADAMTS4 inhibition on cardiac function in pressure-overloaded rat hearts. (*A*) Study design for the testing of ADAMTS inhibitor in AB rats by four groups: (i) sham rats that received vehicle (dark blue); (ii) sham rats that received ADAMTS inhibitor (light blue); (iii) AB rats that received vehicle (red); and (iv) AB rats that received ADAMTS inhibitor (pink). (*B*) Diastolic function assessed by echocardiography; left atrial diameter (left), E/e′ ratio (middle), and diastolic tissue velocity at mitral annulus, i.e. e′ (right). (*C*) Systolic function evaluated in terms of fractional shortening measured by echocardiography (left) and ejection fraction measured by magnetic resonance imaging (right). Representative images for M-mode mid-ventricular recordings (middle). Bars represent mean ± 1 SD. Groups were compared by one-way ANOVA with planned comparisons followed by Bonferroni correction for the following comparisons: sham vehicle vs. sham ADAMTS inhibitor, sham vehicle vs. AB vehicle, and AB vehicle vs. AB ADAMTS inhibitor. *P* < 0.05 are considered significant and marked with *. AB, aortic banding; ADAMTS, a disintegrin and metalloprotease with thrombospondin motif.

The effect of the ADAMTS inhibitor on cardiac function and remodelling was assessed in rat hearts exposed to pressure overload. The rats were placed in four groups: (i) sham rats that received vehicle (no ADAMTS inhibitor), (ii) sham rats that received ADAMTS inhibitor, (iii) AB rats that received vehicle, and (iv) AB rats that received ADAMTS inhibitor (*Figure 1A*). Following AB, echocardiographic examination of vehicle-treated rats revealed a 44% increase in left atrial diameter and a 54% increase in the E/e′ ratio. Both parameters were reduced by close to 30% in rats with AB that received ADAMTS inhibitor. This finding indicated that diastolic function and filling pressures were improved in rats treated with ADAMTS inhibitor (*Figure 1B* and see Supplementary material*online, Table S3*). AB rats that were treated with ADAMTS inhibitor also demonstrated better systolic function than those treated with vehicle (*Figure 1C* and see Supplementary material*online, Table S3*). Signs of advanced heart failure, i.e. the weights of the right ventricles and lungs, were not significantly different in sham compared with AB, or in AB with or without ADAMTS inhibition (see Supplementary material*online, Table S3*). Fewer rats died in the AB group treated with ADAMTS inhibitor (*n* = 7, 29%) than in the vehicle-treated AB group (*n* = 16, 67%), constituting a difference in total mortality [hazard ratio (HR) 0.32 (0.13–0.79), *P* = 0.013, log-rank *P* = 0.008] (see Supplementary material*online, Figure S2* and *Table S4*). We did not observe any clear adverse effects of ADAMTS inhibition (*Supplementary Results*). Overall, cardiac function was better among the surviving AB rats that were treated with ADAMTS inhibitor than among those that received vehicle.

### 3.2 ADAMTS inhibition reduced thickness of left ventricular wall and collagen content in pressure-overloaded rat hearts

**Figure 2:**
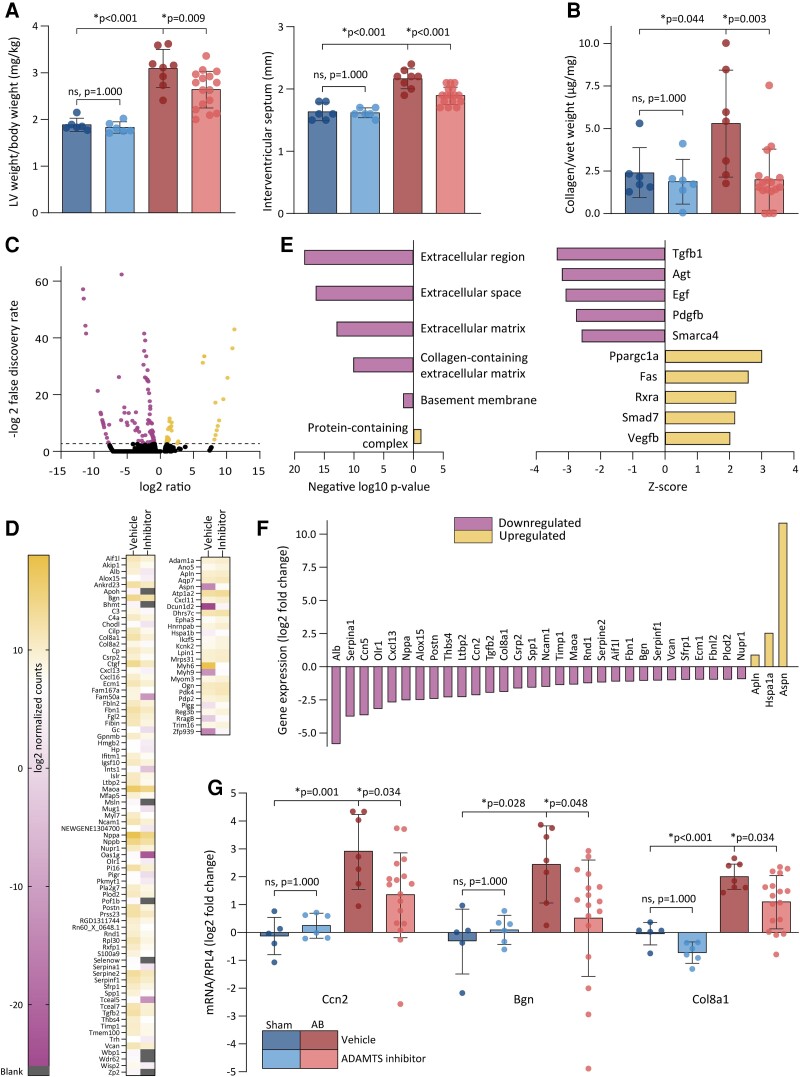
Effects of ADAMTS inhibition on remodelling and disease pathways. (*A*) LV weight to body weight measured at necropsy (left) and interventricular wall thickness measured by echocardiography (right) 8 weeks after AB or sham surgery (sham vehicle *n* = 6, sham ADAMTS inhibitor *n* = 6, AB vehicle *n* = 8, AB ADAMTS inhibitor *n* = 17). (*B*) Fibrosis in LV as determined by the total collagen content as a proportion of wet weight quantified by HPLC. (*C*) Volcano plot showing the expression of genes with a false discovery rate less than 0.15 (black dots), up-regulated (yellow dots), and down-regulated genes (purple dots). Dotted line indicates a false discovery rate of 0.15. (*D*) Heatmap showing log2-transformed normalized counts of DEGs that were down-regulated (left) and up-regulated (right) in AB rats treated with vehicle and ADAMTS inhibitor. Genes with LOC and AARB prefixes are omitted. (*E*) Enrichment of DEGs in cellular compartments assessed by overrepresentation test of genes that were down-regulated (purple) or up-regulated (yellow) in AB rats treated with ADAMTS inhibitor compared with those treated with vehicle (left). Upstream regulators identified in IPA ranked by their *Z*-score (right). (*F*) Gene expression of DEGs that are identified as TGF-β target genes by IPA. (*G*) mRNA expression of selected TGF-β-inducible genes in myocardial samples determined by RT–qPCR (sham vehicle *n* = 5, sham ADAMTS inhibitor *n* = 6, AB vehicle *n* = 7, AB ADAMTS inhibitor *n* = 17). Bars represent mean ± 1 SD. Groups were compared by one-way ANOVA with planned comparisons followed by Bonferroni correction for the following comparisons: sham vehicle vs. sham ADAMTS4 inhibitor, sham vehicle vs. AB vehicle, and AB vehicle vs. AB ADAMTS inhibitor. *P* < 0.05 were considered significant and marked with *. LV, left ventricle; AB, aortic banding; ADAMTS4, a disintegrin and metalloprotease with thrombospondin motif; DEGs, differentially expressed genes; TGF, transforming growth factor; HPLC, high performance liquid chromatography; IPA, Ingenuity pathways analysis; RT–qPCR, real-time quantitative polymerase chain reaction.

To determine whether ADAMTS inhibition affected cardiac remodelling, we assessed cardiac dimensions and myocardial collagen content in the pressure-overloaded rat hearts. In vehicle-treated AB rats, we observed a 64% increase in left ventricular mass and a 34% thicker left ventricular wall. AB rats that were treated with ADAMTS inhibitor exhibited a 15% lower left ventricular weight and a 13% thinner interventricular septum than the AB rats that received vehicle (*Figure 2A* and see Supplementary material*online, Table S3*). The diameter of the left ventricle as measured by echocardiography was lower in the AB rats treated with ADAMTS inhibitor than in AB rats that were treated with vehicle (see Supplementary material*online, Table S3*). AB induced a 2.2-fold increase in collagen content compared with the vehicle-treated sham rats, and both perivascular and interstitial fibrosis were observed in AB rats (see Supplementary material*online, Figure S3*). This collagen amount was reduced in the AB rats treated with ADAMTS inhibitor when compared to AB rats treated with vehicle (*Figure 2B*). Overall, AB rats treated with ADAMTS inhibitor demonstrated reduced left ventricular mass and lower collagen content than AB rats receiving vehicle treatment.

### 3.3 ADAMTS4 inhibition reduced expression of genes associated with TGF-β in pressure-overloaded rats

To identify disease mechanisms targeted by ADAMTS4 inhibition, we performed RNA sequencing of myocardial tissues from both groups of AB rats. In AB rats that had been treated with ADAMTS inhibitor compared with those that had been treated with vehicle, we found 120 DEGs of which 86 genes were down-regulated and 34 were up-regulated in those receiving ADAMTS inhibitor (*Figure2C* and *D* and Supplementary Material). Bioinformatical analyses of the down-regulated genes showed enrichment of ECM-related GO categories (*Figure 2E*), while the central pro-fibrotic factor TGF-β1 was included in the list of inhibited upstream regulators (*Figure 2E*). Since TGF-β is a master regulator of fibrosis,^27^ and 33 downstream target genes of TGF-β1 were altered by ADAMTS4 inhibition (*Figure 2F*), we hypothesized that ADAMTS4 inhibition could reduce the myocardial collagen content through modulation of TGF-β. To verify this finding, we performed real-time quantitative polymerase chain reaction (RT–qPCR) on TGF-β downstream target genes in all four groups. This analysis confirmed that AB rats that had been treated with ADAMTS inhibitor exhibited significant down-regulation of genes that encoded for connective tissue growth factor, biglycan, and collagen 8a1 (*Figure 2G*), all of which are genes implicated in the fibrotic response at similar timepoints post-AB.^19,28^ In contrast, we found no alterations in the expression of selected genes that encode for mediators or markers of hypertrophy or inflammation, which indicated that modulation of these disease processes was not central features of the response to ADAMTS inhibition (see Supplementary material*online, Table S5*). Appreciating the central role of TGF-β in fibrosis and heart failure development after pressure overload,^27^ our results suggested that ADAMTS inhibition prevented fibrosis and cardiac dysfunction by reducing TGF-β-activity.

### 3.4 ADAMTS4 releases ECM-bound latent TGF-β *in vitro*

**Figure 3:**
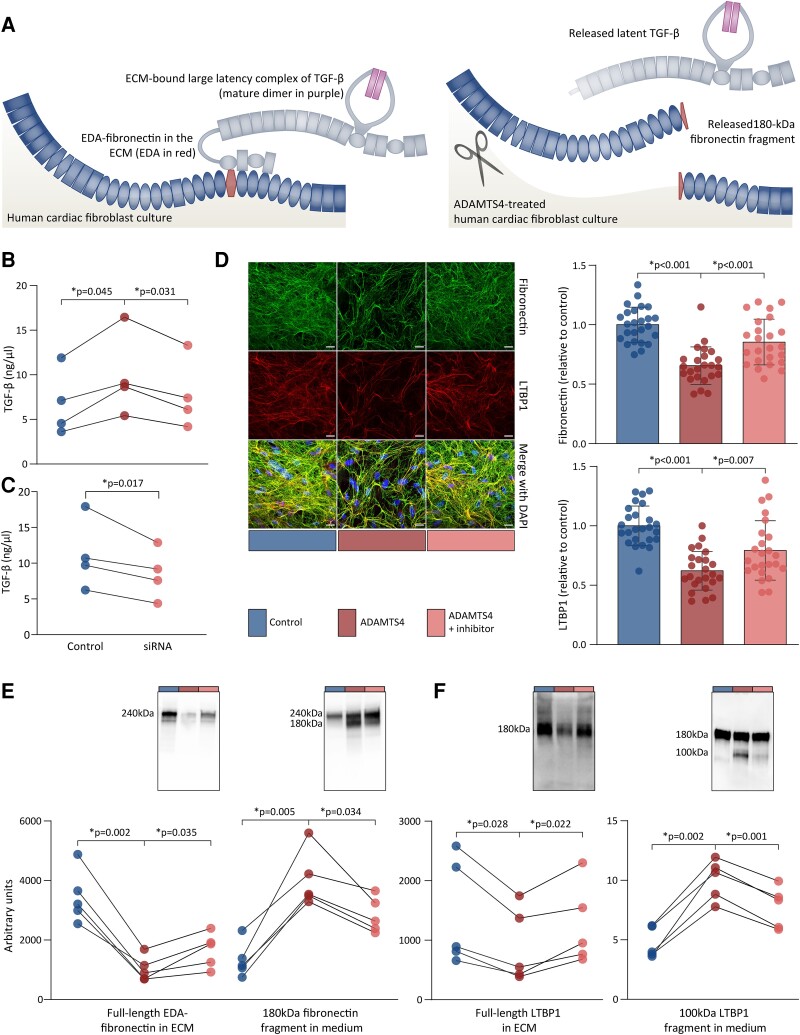
Disruption of TGF-β and its ECM-anchoring proteins by ADAMTS4 in human cardiac fibroblast cultures. Human adult (*B*) or foetal (*C–E*) cardiac fibroblasts were cultured for 7 days and treated with DMSO or ADAMTS4 with or without ADAMTS inhibitor for 24 h. (*A*) Illustration shows how EDA-fibronectin (blue and red) anchors the large latency complex of TGF-β (grey and purple) to the ECM (left). In response to ADAMTS4 (scissor), fibronectin fragments and latent TGF-β are released from the ECM (right). (*B*) Total TGF-β levels in heat-activated media as quantified by tMLC from four independent experiments. (*C*) Total TGF-β levels in heat-activated media as quantified by tMLC from four independent experiments using siRNA to knock down ADAMTS4. (*D*) Representative images of immunofluorescence staining of fibronectin and LTBP1 in the ECM. Area of fibronectin and LTBP1 staining relative to cell number (DAPI) were quantified and normalized to DMSO controls. Data represent quantifications of confocal *Z*-stacks (*n* = 8–9 per experiment) from three independent experiments and the graph bars represent mean ± 1 SD. Scale bars represent 20 μm. (*E*) Immunoblots show full-length EDA-fibronectin (240 kDa) and fragments (180 kDa) in the ECM and medium, respectively. (*F*) LTBP1 full-length (180 kDa) and fragments (100 kDa) in ECM and medium, respectively. Immunoreactive bands were quantified and normalized to total protein levels. Comparisons between groups were assessed by one-way ANOVA for correlated samples (*B*, *D*, and *E*) or planned comparisons (*C*) with Bonferroni correction for multiple testing of controls vs. ADAMTS4 and ADAMTS4 vs. ADAMTS inhibitor. Data points from the same experiment are connected by lines. *P* < 0.05 were considered significant and are marked with *. TGF, transforming growth factor; DMSO, dimethyl sulfoxide; tMLC, transfected mink lung epithelial cells; DAPI, 4′,6-diamidino-2-phenylindole; LTBP, latent TGF-β-binding protein; EDA, extra domain A; ECM, extracellular matrix; ADAMTS4, a disintegrin and metalloprotease with thrombospondin motif.

TGF-β is stored in the ECM as a large latency complex (LLC) that is composed of LTBP1, the latency associated peptide (LAP) and the mature TGF-β-dimer (*Figure 3A*).^29^ To understand and characterize the role of ADAMTS4 in TGF-β signalling, we hypothesized that the cleavage of ECM components by ADAMTS4 could mobilize TGF-β. Thus, we started by determining whether ADAMTS4 could release ECM-anchored latent TGF-β. To adult human cardiac fibroblasts that produced a mature ECM over 7 days,^24^ we added active, recombinant ADAMTS4 with or without inhibitor for 24 h in serum-free medium before analysis. Total TGF-β levels, i.e. of both active and latent forms of TGF-β, were measured in the heat-activated, conditioned medium through the use of tMLC assay. We demonstrated that the treatment with ADAMTS4 increased the level of total TGF-β in the medium by ∼50% (*Figure 3B*), whereas concomitant treatment with ADAMTS inhibitor reduced this release of TGF-β into the medium. Moreover, siRNA-mediated ablation of ADAMTS4 (see Supplementary material*online, Figure S4*) reduced the levels of total TGF-β in the medium in cultures of human cardiac fibroblasts (*Figure 3C*). These observations suggest that latent TGF-β is transferred from the ECM to the medium upon treatment with ADAMTS4.

### 3.5 ADAMTS4 disrupts organisation of TGF-β-binding ECM components

We next sought to understand the mechanism of ADAMTS4-mediated release of ECM-bound latent TGF-β. Interactions between LTBP1 and ECM components promote TGF-β latency,^30^ as assembly of the LLC and anchorage to the ECM depends on LTBP1, LAP, and fibronectin.^31,32^ Thus, we anticipated that the cleavage of TGF-β-binding ECM components, such as LTBP1 and fibronectin, might mobilize latent TGF-β. LTBP1 binds the ECM glycoprotein fibronectin through a binding site within the fibronectin type III domains 12–14,^33^ supported by an additional binding site in the EDA when this isoform called EDA-fibronectin is present.^34^ Since it was a potential mechanism for TGF-β release, we determined the effect of ADAMTS4 on LTBP1 and fibronectin in the ECM *in vitro*. In the ECM of foetal human cardiac fibroblasts, we observed a 34% reduction in the amount of fibronectin and a 38% reduction in the amount of LTBP1 upon ADAMTS4 treatment, as assessed by immunohistochemistry (*Figure 3D*). To determine whether the losses of ECM-localized fibronectin and LTBP1 were associated with proteolysis, we assessed the effects of ADAMTS4 on full-length proteins and potential cleavage fragments by immunoblotting. In ECM lysates from ADAMTS4-treated foetal human cardiac fibroblasts, we observed a 70% reduction in the amount of full-length EDA-fibronectin (*Figure 3E*) and a 39% reduction in the amount of full-length LTBP1 (*Figure 3F*). Also, ADAMTS4 knockdown reduced the amount of LTBP1 in the ECM (see Supplementary material*online, Figure S4*). Meanwhile, fragments of these components appeared in the medium upon treatment with ADAMTS4. An 180 kDa fibronectin fragment of fibronectin and a 100 kDa fragment of LTBP1 were observed in the conditioned media after ADAMTS4 treatment, while not being clearly present in controls (*Figure 3F*). Concomitant treatment with ADAMTS inhibitor counteracted the effects of ADAMTS4, reducing the loss of fibronectin and LTBP1 from the ECM and the release of fragments to the media (*Figure3B*, *D*, *E*, and *F*). These observations indicate that ADAMTS4 disrupts EDA-fibronectin and LTBP1 in the ECM through enzymatic cleavage.

### 3.6 ADAMTS4 cleaves LTBP1 and EDA-fibronectin

**Figure 4:**
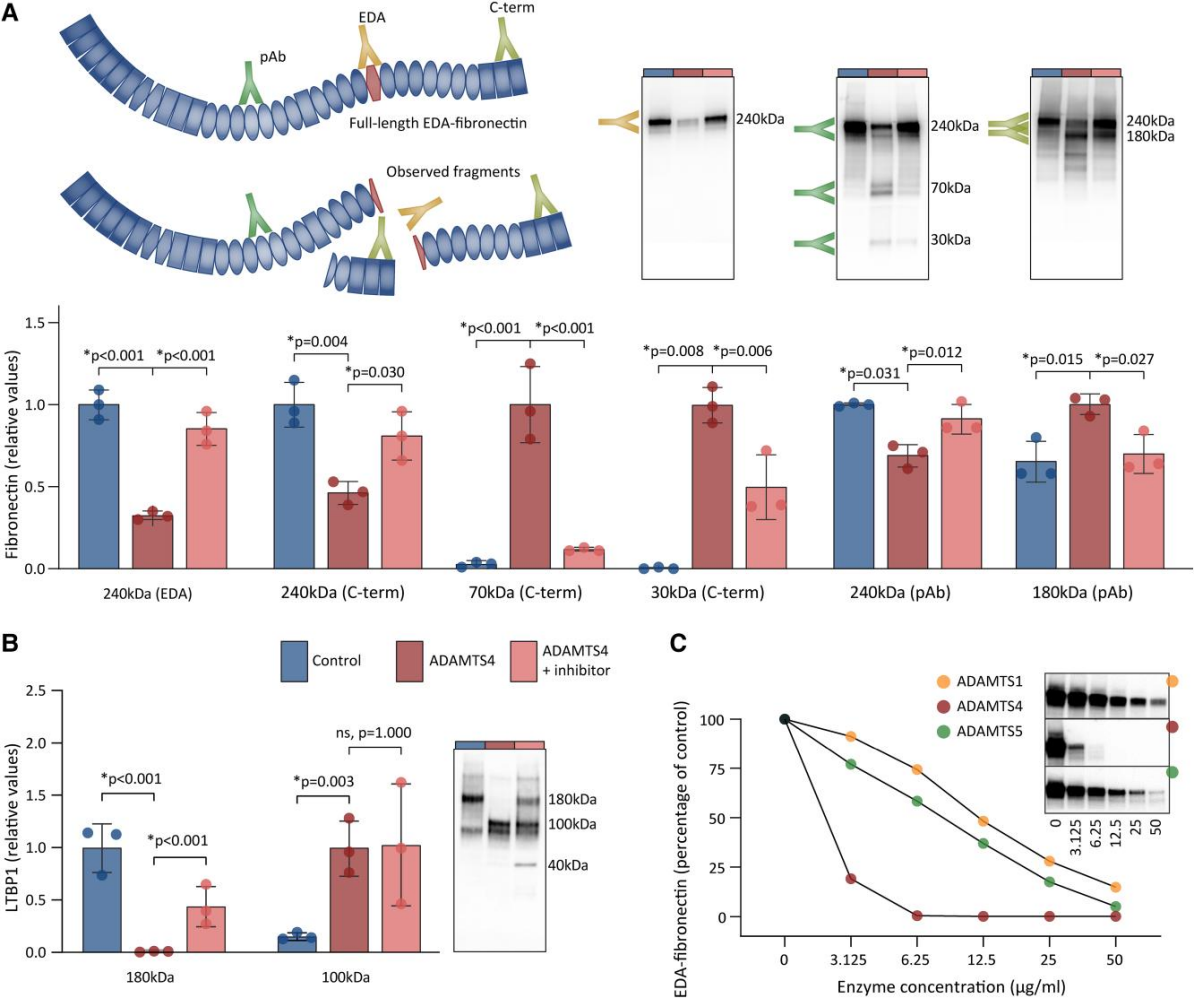
ADAMTS4-mediated cleavage of EDA-fibronectin and LTBP1. Human cardiac fibroblasts were cultured for 7 days, before lysates were treated with DMSO or ADAMTS4 with or without ADAMTS inhibitor. (*A*) Fibronectin cleavage assessed by antibodies specific for EDA and C-terminus (C-term), in addition to a polyclonal antibody (pAb). Relative values to control (full-length protein) or to ADAMTS4-treated lysates (fragments) are shown. Representative immunblots shown. Illustration shows the fibronectin fragments that were detected by the three antibodies. Cleavage sites were as indicated by the size of the fragments and antibody epitopes. (*B*) Representative immunoblot and quantification showing LTBP1 cleavage by ADAMTS4. (*C*) Concentration response of fibronectin cleavage by ADAMTS4 (red) in comparison with ADAMTS1 (yellow) and -5 (green), determined by the amount of full-length EDA-fibronectin detected by EDA-specific antibody in response to increasing enzyme concentrations. Immunoreactive bands were normalized to total protein levels. Data represent quantifications from three independent experiments, and the graph bars represent mean ± 1 SD (*A* and *B*). Comparisons between groups assessed by one-way ANOVA with Bonferroni correction for the following comparisons: control vs. ADAMTS4; and ADAMTS4 vs. ADAMTS inhibitor. For 70 kDa C-terminal fibronectin fragment and LTBP1, log2-transformed values are used. *P* < 0.05 were considered significant and are marked with *. DMSO, dimethyl sulfoxide; EDA, extra domain A; LTBP, latent TGF-β-binding protein; ADAMTS4, a disintegrin and metalloprotease with thrombospondin motif.

To confirm that the release of fibronectin and LTBP1 fragments was due to direct cleavage by ADAMTS4, we next incubated foetal human cardiac fibroblast lysates with ADAMTS4 alone or in combination with the ADAMTS inhibitor. To characterize the cleavage fragments at fibronectin immunoblots, we used antibodies that recognized the EDA and C-terminus, in addition to a polyclonal antibody that detected other parts of the protein (*Figure 4A*). In ADAMTS4-treated lysates, we observed a reduction in the amount of full-length fibronectin, as detected by all three antibodies, and this reduction was blunted by the addition of the ADAMTS inhibitor. Three cleavage fragments of fibronectin were observed after treatment with ADAMTS4, which were two C-terminal fragments of 30 and 70 kDa, and an 180 kDa fragment recognized by the polyclonal antibody (*Figure 4A*). No fragments were detected through the use of the EDA-specific antibody. The size of the fragments indicated cleavage within or close to the EDA. This supposition was supported by additional observations in a smaller fibronectin peptide (see Supplementary material*online, Figure S5*). For LTBP1, we observed a loss of full-length protein and appearance of a 100 kDa fragment in ADAMTS4-treated lysates, while only the loss of full-length protein was prevented by the inhibitor (*Figure 4B*). Taken together, these results indicate that ADAMTS4 cleaves EDA-fibronectin and LTBP1, and that EDA-fibronectin cleavage is blunted by treatment with ADAMTS inhibitor.

### 3.7 ADAMTS4 cleaves EDA-fibronectin more efficiently than other ADAMTS enzymes

To determine whether the cleavage of EDA-fibronectin is specifically due to ADAMTS4 activity, we assessed the concentration–response relationship for ADAMTS4 and similar enzymes. Since ADAMTS1, -4, and -5 share proteolytic activities,^35^ we compared the efficacy of these enzymes in the cleavage of EDA-fibronectin. ADAMTS4 cleaved EDA-fibronectin at lower enzyme concentrations than ADAMTS1 or -5 (*Figure 4C*). An addition of the ADAMTS inhibitor reduced the amount of EDA-fibronectin cleavage by all three enzymes at an inhibitor concentration equal to or greater than 100 nM (see Supplementary material*online, Figure S6A*). Versican cleavage was affected both by ADAMTS4 or -5, whereas the ADAMTS inhibitor prevented the versican cleavage by ADAMTS4, but did not prevent this action by ADAMTS5 (see Supplementary material*online, Figure S6B* and *C*).

### 3.8 Increased ADAMTS4 activity and effects of ADAMTS4 inhibition in pressure-overloaded rat hearts

**Figure 5:**
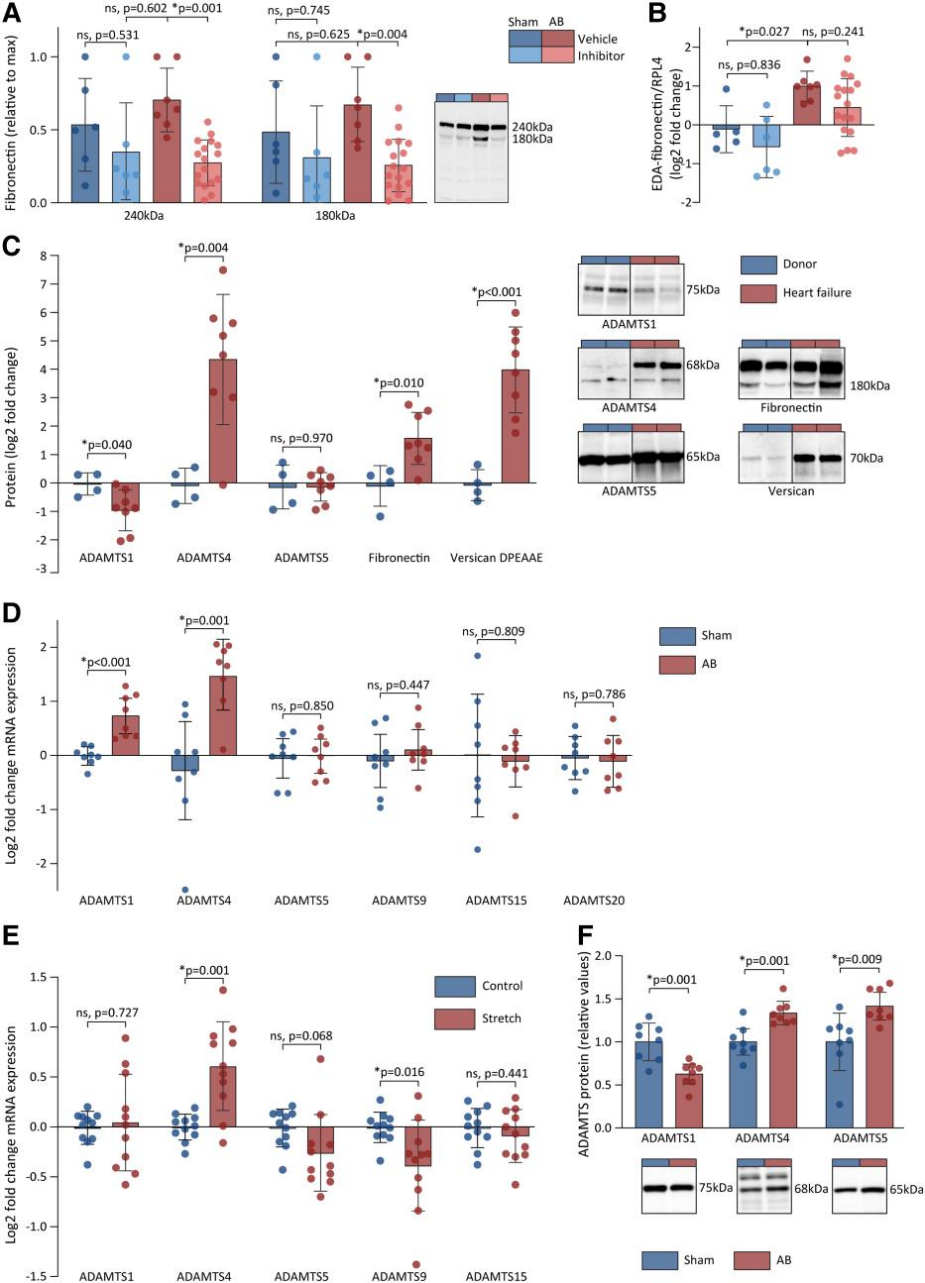
ADAMTS4 activity is increased in the myocardium of rats and patients with cardiac dysfunction. (*A*) The myocardial amount of full-length fibronectin (240 kDa) and its cleavage fragment (180 kDa) as determined by immunoblots in sham vehicle (*n* = 6), sham ADAMTS inhibitor (*n* = 6), AB vehicle (*n* = 7), and AB ADAMTS inhibitor (*n* = 17). Representative blots shown. (*B*) mRNA levels of EDA-fibronectin in myocardial samples from AB rats determined by RT–qPCR in sham vehicle (*n* = 5), sham ADAMTS inhibitor (*n* = 6), AB vehicle (*n* = 7), and AB ADAMTS inhibitor (*n* = 17). (*C*) The levels of active ADAMTS1, -4, and -5 proteins, fibronectin 180 kDa fragments, and versican DPEAAE fragments in myocardial samples from explanted human failing hearts (red) compared with healthy donor hearts (blue). Representative blots shown for ADAMTS4 (left), EDA-fibronectin (middle), and versican DPEAAE fragments (right). (*D*) mRNA levels of ADAMTS1, -4, -5, -9, -15, and -20 in myocardial samples from rats 6 weeks after AB (*n* = 8) or sham (*n* = 8). (*E*) Log2-transformed mRNA levels of ADAMTS1, -4, -5, -9, and -15 in adult human cardiac fibroblasts exposed to stretch (*n* = 11) compared to control conditions (*n* = 11). ADAMTS20 levels were not detectable. (*F*) The levels of ADAMTS1, -4, and -5 protein in myocardial lysates from rats 6 weeks after AB (*n* = 8) or sham (*n* = 8) relative to sham group. Bars represent mean ± 1 SD. Groups were compared by one-way ANOVA with planned comparisons followed by Bonferroni correction for the following comparisons: sham vehicle vs. sham ADAMTS inhibitor, sham vehicle vs. AB vehicle, and AB vehicle vs. AB ADAMTS inhibitor. Groups were compared by Student’s *t*-test for the following comparisons: sham vs. AB, control vs. stretched cells, and donor vs. heart failure patients. *P* < 0.05 were considered significant and marked with *. AB, aortic banding; EDA, extradomain A; ADAMTS4, a disintegrin and metalloprotease with thrombospondin motif; RT–qPCR, real-time quantitative polymerase chain reaction; DCM, dilated cardiomyopathy.

Having demonstrated that ADAMTS4 cleaved fibronectin *in vitro*, we sought to explore whether this mechanism also occurred *in vivo*. Fibronectin cleavage was determined by immunoblotting of ECM-rich protein fractions from rat cardiac tissues. Similar to our *in vitro* findings, we observed an accumulation of the 180 kDa fragment of fibronectin in AB rats. This level was reduced when ADAMTS inhibitor was present (*Figure 5A*). In addition, an increase in the mRNA expression of EDA-fibronectin following AB (*Figure 5B*) supported the assumption that the EDA-fibronectin isoform was available in rat myocardium that was exposed to pressure overload. The myocardial ADAMTS activity in AB rats was confirmed by the accumulation of versican DPEAAE fragments, the production of which was reduced when ADAMTS inhibitor was present (see Supplementary material*online, Figure S7C*). A non-significant increase of the 100 kDa fragment of LTBP1 was observed in vehicle-treated AB compared to sham rats, with a non-significant reduction following treatment with ADAMTS inhibitor (see Supplementary material*online, Figure S8*).

### 3.9 ADAMTS4 activity is increased *in vivo*

To determine whether myocardial ADAMTS4 activity is increased during human heart failure, we assessed the level of active ADAMTS4 protein and its cleavage products in explanted hearts with dilated cardiomyopathy (DCM) (see Supplementary material*online, Table S6*). Compared with healthy donor hearts, a 3.5-fold increase in the amount of 180 kDa fibronectin in the non-ischaemic failing hearts was observed (*Figure 5C*). A 24-fold increase in the amount of versican DPEAAE fragments in non-ischaemic failing hearts was observed, whereas these fragments were present in negligible amounts in donor hearts (*Figure 5C*). A non-significant increase in the 100 kDa fragment of LTBP1 was observed in non-ischaemic failing hearts (see Supplementary material*online, Figure S8*). Taken together, these data indicate that ADAMTS4 activity, including fibronectin cleavage, is increased in human heart failure.

### 3.10 ADAMTS4 levels are enhanced by mechanical stress and in human heart failure

Since ADAMTS1, -5, -9, -15, or -20 possess similar enzymatic activities as ADAMTS4, we examined their expression pattern in pressure-overloaded rat hearts, cardiac fibroblasts exposed to mechanical stress, and in failing human hearts. The amounts of ADAMTS1 and -4 mRNA were up-regulated 6 weeks after AB (*Figure 5D* and see Supplementary material*online, Table S7*), while only ADAMTS4 was up-regulated in human cardiac fibroblasts exposed to mechanical stress (*Figure 5E*). The amount of 68 kDa ADAMTS4 protein, consistent with the mature form of the enzyme,^36^ was increased in AB rats and DCM patients, and ADAMTS5 protein was increased in AB rats, while ADAMTS1 levels were decreased in both AB rats and DCM patients (*Figure5C* and *F*). To summarize, the increased levels of ADAMTS4 seem to be consistent across different patient cohorts and models related to heart failure and cardiac fibrosis.

## 4. Discussion

In this study, we have demonstrated that (i) ADAMTS inhibition alleviates cardiac dysfunction and reduces myocardial collagen content in pressure-overloaded rat hearts; (ii) ADAMTS4 releases ECM-bound latent TGF-β partly by cleaving TGF-β-anchoring EDA-fibronectin; and (iii) ADAMTS4 is up-regulated and active in human and murine failing hearts. Taken together, these findings indicate that ADAMTS4 is a central mediator of cardiac fibrosis, and therefore, it may be a target for the treatment of patients with cardiac fibrosis and heart failure (*Graphical abstract*).

Our findings demonstrate marked improvement of cardiac function caused by using an ADAMTS inhibitor with a high potency for inhibiting ADAMTS4, and also a novel mechanism for the beneficial effects on cardiac remodelling. The AB model recapitulates the extensive fibrotic response to increased pressure overload.^37^ Since fibrosis is an essential cause of myocardial stiffness,^38^ the anti-fibrotic effect of ADAMTS inhibition may explain the beneficial effects of such inhibition on cardiac diastolic dysfunction. Based on transcriptional changes, we have identified pro-fibrotic regulators that are affected by the ADAMTS inhibitor, particularly TGF-β1. TGF-β governs differentiation of fibroblasts into ECM-producing myofibroblasts, and therefore promotes production of excessive ECM and serves as a critical regulator of cardiac remodelling in response to pressure overload.^37^ Our findings indicate that an effect of inhibiting ADAMTS4 activity in pressure overload lies in TGF-β suppression and subsequent reductions in fibrosis.

In human cardiac fibroblast cultures with a mature ECM, we have identified ADAMTS4 as a regulator of latent TGF-β anchorage to the ECM, thus presenting an explanation for the effects observed *in vivo*. The ECM-bound latent TGF-β constitutes a reservoir that can be rapidly mobilized.^39^ Proteolytic activation, among several activation mechanisms for TGF-β, denotes proteolytic release of ECM-bound latent TGF-β, which is then made available for membrane-localized activators.^40–43^ Binding of TGF-β to LTBP1 maintains latency, and LTBP1 is anchored to ECM through its interaction with fibronectin.^44^ This interaction is facilitated by the EDA domain of fibronectin.^34^ We have demonstrated an enrichment of EDA-fibronectin in cardiac ECM after pressure overload *in vivo*. Previous reports have demonstrated that EDA-fibronectin is involved in cardiac fibrosis^45,46^ and serves as a central regulator of TGF-β latency in other tissues^47^ and cell cultures.^34,48,49^ As such, the simultaneous disruption of EDA-fibronectin and release of latent TGF-β upon treatment with ADAMTS4 in cultured human cardiac fibroblasts provide a robust indication of a causal relationship between fibronectin cleavage and mobilisation of ECM-stored TGF-β. The hypothesis that this mechanism is relevant *in vivo* is supported by the presence of the same cleavage products of fibronectin in failing human and pressure-overloaded rat hearts, as observed in our *in vitro* models.

We demonstrate for the first time that ADAMTS4 cleaves fibronectin within the EDA domain. This finding has implications for the downstream effects. Although other proteases are known to cleave fibronectin,^50,51^ direct cleavage of the EDA has not been reported. For instance, ADAMTS2, -3, and -14 are reported to cleave fibronectin at a site outside EDA.^52^ The presence of an additional 30 kDa C-terminal fragment of fibronectin suggests a second cleavage of the C-terminal to the heparin-binding domain, which could correspond to a reported cleavage site susceptible to cleavage by ADAMTS16.^53^ Since EDA-fibronectin anchors LTBP1 through interactions at its EDA and heparin-binding domains, the ADAMTS4-mediated cleavage within the EDA and at the C-terminal side of the heparin-binding domain could contribute to the detachment of LTBP1 from the remaining EDA-fibronectin protein. The action of ADAMTS4 also released a 100 kDa fragment of LTBP1. However, the effect of the ADAMTS inhibitor to prevent the release of LTBP1 fragments in human cardiac fibroblast cultures was not observed in lysates and not observed *in vivo*, which may indicate that LTBP1 is primarily cleaved after its release from fibronectin. Indeed, the presence of LTBP1 fragments of similar sizes is suggested to indicate that a protease-resistant core domain is found between protease-sensitive sites,^40^ which could be more exposed once it is detached from the ECM. Combined with the extensive effects on fibronectin disruption in human cardiac fibroblast cultures, and the presence of fibronectin fragments in remodelled human and rat hearts, we consider that cleavage of EDA-fibronectin is the main effect of ADAMTS4 in its mobilisation of latent TGF-β.

Although ADAMTS enzymes are considered to have a narrower substrate repertoire than other ECM-proteolytic enzymes,^35^ fibronectin and LTBP1 are not the only substrates of potential relevance in heart failure. ADAMTS4 cleaves versican to generate DPEAAE fragments. We observed increased amounts of this fragment in the pressure-overloaded myocardium and in human cardiac fibroblast cultures that had been treated with ADAMTS4. It has been demonstrated that the presence of this fragment in other tissues facilitates immune cell infiltration and induces immune responses.^54,55^ In addition, a build-up of versican and ECM due to lack of proper ADAMTS-mediated degradation is a suggested contributor to cardiac fibrosis^56^ and aortic fibrosis.^57^ However, our phenotyping and transcriptome analyses indicated that fibrosis is reduced, and inflammatory processes are less affected in the treated pressure-overloaded rat hearts.

ADAMTS4 belongs to a group of ADAMTS enzymes that are characterized by their ability to cleave proteoglycans. The proteoglycanases also include ADAMTS1, -5, -9, -15, and -20.^58^ Although these enzymes have overlapping substrate repertoires, their expression patterns depend on tissue and condition, where ADAMTS1, -4, and -5 are implicated in cardiac remodelling,^14,15,16,56,59^ and ADAMTS4 is induced in response to mechanical stress and other pathological stimuli.^14,15,16^ Despite sharing cleavage activities, different roles of these enzymes have been observed in aortic dilatation, where knockouts of ADAMTS1^60^ and -5^61^ are deleterious, and ADAMTS4 knockout protective.^62^ Similarly, in pressure overloaded murine models, ablation of ADAMTS5 catalytic site worsens heart function,^56^ as opposed to our observations in response to the tested inhibitor with a high potency for ADAMTS4 inhibition. Loss-of-function studies in ADAMTS4 knockout models could further elucidate the distinct roles of ADAMTS4.

Although we have previously observed beneficial effects of the inhibitor pentosane polysulfate in AB rats,^16^ the use of a more selective inhibitor of ADAMTS enzymes in this study supports the potential for ADAMTS4 as a therapeutic target. In line with previous reports,^63^ ADAMTS1, -4, and -5 produced the versican DPEAAE fragment. While the presence of the ADAMTS inhibitor most likely did not inhibit ADAMTS5 versicanase activity at the doses employed in our study, a concomitant effect on ADAMTS1 may be present. However, the versicanase activity of ADAMTS1 is weak, as also noted in other studies *in vitro*^64^ and *in vivo*.^61^ Although the ADAMTS inhibitor prevented fibronectin cleavage exerted by all tested ADAMTS enzymes, we demonstrated that fibronectin was more efficiently cleaved by ADAMTS4. Therefore, our data support the targeting of ADAMTS4 as a means to prevent cardiac fibrosis and diastolic dysfunction and indicate that fibronectin cleavage could be mechanistically involved in this beneficial effect.

Our findings should be interpreted in light of the limitations of the study. First, although we did not observe any adverse effects of ADAMTS4 inhibition in our study, infrequent events or off-target effects cannot be excluded. However, repeated administration of a similar compound did not lead to adverse effects in guinea pigs.^17^ Although the animals received optimal care throughout the study, stressors such as daily oral gavage and anaesthesia could have contributed to a high mortality rate and the subsequent low number of animals in the vehicle-treated AB group. Therefore, the statistical power may have been too limited to detect some effects of treatment, such as a significant reduction in myocardial expression of natriuretic peptides, whereas the effects that were observed should be robust and reliable. Differences in heart rate during echocardiographic examination could have influenced recordings on heart function. The beneficial effects observed in our study may also be influenced by effects on non-myocardial tissues. Of note, ADAMTS enzymes are implicated in aortic remodelling and blood pressure regulation.^60–62^ Although no gross changes in aortic structure were observed in AB rats treated with ADAMTS inhibitor (see Supplementary material*online, Table S3*) or have been reported in unchallenged ADAMTS4 KO mice,^65,66^ exposing the vasculature to stress during AB may affect aortic remodelling. Therefore, future studies on the roles of ADAMTS4 in cardiac disease should include blood pressure measurements and aortic examinations.

Fibrosis is a central characteristic of heart failure of different phenotypes and aetiologies,^67^ and TGF-β represents an attractive therapeutic target. However, direct inhibition of TGF-β-signalling has proved to have ambiguous effects in rats exposed to pressure overload due to detrimental effects on wound healing.^68^ Therefore, approaches to inhibit the detrimental actions of TGF-β without interfering with its physiological roles are warranted.^69^ The findings that ADAMTS4 is up-regulated in heart failure and that it cleaves EDA-fibronectin, which is an ECM component mainly seen in diseased tissue, strengthen the idea that ADAMTS4 should be a specific target in TGF-β modulation and anti-fibrotic treatment in heart disease. Although fibrosis occurs in most forms of heart failure,^67^ the activity of ADAMTS4 in other phenotypes than those examined in our study should be clarified to further support the translational potential of these findings.

## Supplementary material

Supplementary material is available at *Cardiovascular Research* online.

## Authors’ contributions

Conceptualization: M.V., P.M.E., A.S., A.R., C.R.C., J.M.A., I.S., G.C. Methodology: M.V., P.M.E., A.S., E.S.N., K.B., A.R., A.O.M., J.M.A., I.S., G.C. Validation: M.V., P.M.E., A.S., A.R., I.G.L., L.Z., M.B.Ø., J.Ø. Formal analysis: M.V., P.M.E., I.G.L. Investigation: M.V., P.M.E., A.S., E.S.N., K.B., A.R., I.G.L., L.Z., M.B.O., J.Ø., C.R.C., C.H.W., J.R., C.P.D., A.E.F., I.M.H.I., E.E., A.O.M., T.T., J.M.A., I.S., G.C. Resources: M.V., E.S.N., K.B., L.Z., C.H.W., J.R., C.P.D., A.E.F., I.M.H.I., E.E., T.T., J.M.A., I.S. Writing—original draft: M.V., G.C., P.M.E. Writing—review & editing: all. Visualization: M.V.

Supervision: M.V., G.C. Project management: M.V., G.C. Funding acquisition: M.V., G.C.

## Supplementary Material

cvad078_Supplementary_Data

## Data Availability

Data underlying this article is available in the article and its online Supplementary Material. In addition, all raw data are archived at the Institute for Experimental Medical Research and available upon request, with the exception of patient identifiable data.

## Notes

Translational perspective In this study, we have identified the extracellular matrix enzyme A disintegrin and metalloprotease with thrombospondin motif 4 (ADAMTS4) as a promising target for protection against heart failure and cardiac fibrosis. In pressure-overloaded rat hearts, treatment with an inhibitor of ADAMTS enzymes prevented development of cardiac fibrosis and dysfunction. A marked increase in ADAMTS4 expression and activity in failing human hearts supports a translational potential for this treatment principle. The findings are particularly promising in light of the urgent need for novel and innovative therapies in heart failure subpopulations characterized by cardiac fibrosis.
