# Supplementary material for: Inhibition of the extracellular enzyme A disintegrin and metalloprotease with thrombospondin motif 4 prevents cardiac fibrosis and dysfunction
Source: Cardiovasc Res. 2023 May 22;119(10):1915–27. doi: 10.1093/cvr/cvad078 (PMC10439713; doi:10.1093/cvr/cvad078)
Supplement: cvad078_Supplementary_Data [file cvad078_supplementary_data.docx]

**Supplementary Methods**

**Pharmacokinetic study and dose simulations**

Previous pharmacokinetic studies of the ADAMTS inhibitor had demonstrated a half-maximal inhibitory concentration (IC_50_) of 26nM, and led to a prediction of a human dose of 7-126mg/day b.i.d.,^1^ equivalent to 0.62-11.16mg/kg/day in rats.^2^ AstraZeneca detected a maximum tolerated dose at 500mg/kg and plasma protein binding of 0.5103, and assessed the pharmacokinetic properties of the ADAMTS inhibitor in Wistar rats based on plasma levels after an oral administration of 2mg/kg of ADAMTS inhibitor as a suspension. A half-life of 3.3 hours after a 2mg/kg dose in rats was demonstrated (personal communication Pär Nordell at AstraZeneca). Based on the pharmacokinetic properties, dose simulations of higher doses were performed to determine the appropriate dose that would yield sufficient efficacy and selectivity.

**Aortic banding (AB) rat model for *in vivo* testing of ADAMTS4 inhibition**

A sample size calculation revealed that 17 animals were required in each group to detect a similar change in lung weight and fractional shortening, as was demonstrated for PPS in our previous study^3^ with a power of 0.85. Based on an estimated mortality rate of 30% after AB, 24 animals were placed in the AB groups. After surgery, the animals were inspected daily by a member of staff who was blinded to the treatment groups. Bedding was changed weekly. Animals that died during the treatment period underwent necropsy to assess the cause of death.

**Randomisation to treatment groups**

At day 1 post-operatively, we assessed the post-AB cardiac dysfunction based on left atrial diameter, and separated the rats into strata that ranged from 3 to 6mm, with 0.5mm in each stratum (3.0-3.5, 3.6-4.0, 4.1-4.5, 4.6-5.0, 5.1-5.5, 5.6-6.0mm). Thereafter, we paired the animals within each stratum or the closest stratum, and randomised them by coin toss to treatment with either ADAMTS inhibitor or vehicle. No difference in cardiac dimensions was observed at randomisation (Table S1).

**Preparation of microsuspension and administration of ADAMTS inhibitor**

The ADAMTS4 inhibitor was mixed in 5mg/g microsuspension. In brief, ADAMTS inhibitor was diluted in a vehicle solution containing 0.1% 20mM Tween-80 and 0.5% hydroxypropylmethylcellulose (HPMC) in distilled H_2_O. The microsuspension was left on magnetic stirring overnight to micronise the compound to fine particles, and then stored at -70^o^C until it was required for administration. The vehicle that contained Tween-80 and HPMC was used as the control treatment. The frozen suspension was thawed at 4^o^C for 24 hours before administration. To ensure homogeneous suspension before administration, the suspension was mixed immediately before gavage by ultrasonication at 23^o^C for 10 minutes, followed by mixture by magnetic stirrer for 15 minutes. Visual inspection ensured that it constituted a homogeneous suspension with no precipitate. The suspension was left on the magnetic stirrer during administration. The rats were weighed weekly to calculate the amount of microsuspension or vehicle solution that should be administered.

**Echocardiography**

Echocardiography was performed by experienced researchers (JMA for *in vivo* testing of ADAMTS inhibition and IS for ADAMTS expression analyses), who were blinded to groups, using the VEVO 2100 system (VisualSonics, Canada). During the examination, rats were sedated through supply of 2% isoflurane and 98% oxygen via a mask. The left ventricular inner diameter in systole (LVIDs) and diastole (LVIDd), and thicknesses of the interventricular septum (IVDd) and posterior wall (PWDd) in diastole were measured to assess left ventricular dimensions. Left ventricular systolic performance was assessed through measurements of fractional shortening and peak systolic tissue velocity, whereas diastolic function and increased filling pressures were evaluated by the peak mitral inflow velocity (E-wave) and diastolic tissue velocity measure corresponding to e’, as well as left atrial diameter.

**Harvesting and necropsy**

The hearts excised from the rats were rinsed in phosphate-buffered saline (PBS) for *in vivo* testing of ADAMTS inhibition and in 0.9% NaCl for the ADAMTS expression analyses. The hearts were weighed before and after the right ventricle was separated from the left. Tissue was frozen in liquid nitrogen and stored at -70^o^C.

**AB rat model for expression analyses**

Rats were randomized for sham surgery (n=8) and AB (n=8). AB was performed using an o-ring with fixed inner diameter of 1.07 mm around the ascending aorta, by one experienced rat surgeon (AOM). Animals received operative analgesics by subcutaneous injection of buprenorphine pre-surgery and every 8 hours post-surgery for 24 hours. Rats were hosted in cages of 2-3 animals per cage, 12h/12h light-dark cycle and *ad libitum* access to food and water.

**Quantification of collagen content by HPLC and Masson’s trichrome staining**

Total collagen content as a proportion of the wet weight was calculated by HPLC, according to the manufacturer’s protocol (AccQ-Fluor Reagent Kit WAT052880, Waters, MA) and as described previously.^4^ Formalin-fixed paraffin-embedded transverse sections of left ventricle were stained with Masson’s trichrome staining (Polysciences Inc, PA) according to manufacturers’ instructions. The whole sections were imaged using an Axioscan Z1 (Carl Zeiss, Germany) and images were processed using Zen (Carl Zeiss) and Fiji/ImageJ.

**Tissue lysates from the left ventricle for immunoblotting**

Protein from frozen left ventricles was extracted to obtain ECM-rich fractions through the application of a compartment protein extraction kit (2145, Millipore, MA) according to the manufacturer’s instructions and the insoluble lysates were used to extract ECM fractions as has been previously described.^5^ For fibronectin and LTBP1 in rats, and for human samples, ECM-rich fractions were prepared. For versican DPEAAE fragments in rats, a three-step protocol described previously was employed.^6^ After removal of NaCl- and SDS-soluble extracts, the lysates were treated with 4M guanidine-HCl, 50mM sodium acetate of pH 5.8 and 25mM ethylenediaminetetraacetic acid with protease and phosphatase inhibitors to obtain ECM-rich extracts. The following antibodies were used for ADAMTS detection: ADAMTS1 (ab236497, Abcam, UK), ADAMTS4 (ab185722, Abcam, UK) and ADAMTS5 (ab41037, Abcam, UK). Versican fragments were detected by a DPEAAE specific antibody (ab19345, Abcam, UK). For detecting fibronectin and LTBP1 in human samples, ab2413 (Abcam, UK) and mAb 388 (R&D systems) antibodies were used respectively.

**RNA-isolation and qPCR**

RNA was extracted from frozen left ventricles and cell cultures using the RNeasy mini kit (Qiagen, Germany), while cDNA synthesis was performed using the iScript cDNA synthesis kit (BioRad, CA). Gene expression was determined through use of q-PCR that employed predesigned, validated TaqMan assays (Ccn2 Rn01537279_g1, Bgn Rn01529736_m1, Col8a1 Rn07316403_m1, Adamts1 Rn01646120_g1, Adamts4 Rn02103282_s1, Adamts5 Rn01458486_m1, Adamts9 Rn01425216_m1, Adamts15 Rn01524703_m1, Adamts20 Rn01407540_m1, Nppb Rn00580641_m1, Acta1 Rn01426628_g1, Myh6 (MHC-α) Rn00691721_g1, Myh7 (MHC-β) Rn00568328_m1, Tnfa Rn99999017_m1, Il1b Rn00580432_m1, Il6 Rn01410330_m1, Ccl2 Rn01456716_g1, Rpl4 Rn00821091_g1) and custom-made assay for ADAMTS4 (APCFDEP). In RNA from human cardiac fibroblasts, predesigned, validated Taqman assays were used (Adamts1 Hs00199608_m1, Adamts4 Hs00192708_m1, Adamts5 Hs01095518_m1, Adamts9 Hs00172025_m1, Adamts15 Hs00373520_m1, Adamts20 Hs00228033_m1).

**RNA sequencing**

For RNA sequencing, we selected three samples from each group of AB rats that were closest to the mean of their group in terms of LV mass, and within two standard deviations of the mean for ejection fraction, left atrial diameter, lung weight and level of fibrosis. RNA sequencing was performed on a HiSeq 4000 machine by paired-end sequencing with a read length of 150bp (H4, Hx). RNA sequencing data were analysed through use of Salmon to map reads to the reference genome, using default parameters.^7^ As reference genome, we used the Ensembl annotation based on the genome build rn6, found at ftp://ftp.ensembl.org/pub/release-90/fasta/rattus_norvegicus//cdna/Rattus_norvegicus.Rnor_6.0.cdna.all.fa.gz. We performed a gene-centric analysis, in which normalisation between samples was employed using the trimmed mean of M values function of the edgeR R/bioconductor library on the number of reads per gene.^8^ To calculate the false discovery rate, we used the estimateGLMTrendedDisp function of the edgeR library, using default parameters.

**Cultures of human cardiac fibroblasts**

Primary foetal human cardiac fibroblasts (306-05F, Cell Applications Inc, CA) were cultured at 37°C in cardiac fibroblast growth medium (316-500, Cell Applications Inc, CA) with 1% penicillin-streptomycin (PEST, Sigma-Aldrich, MO) according to the manufacturer’s instructions. Adult human cardiac fibroblasts (C-12375, PromoCell, Germany) were cultured at 37°C in PromoCell fibroblast growth medium 3 (C-23025, PromoCell, Germany) with 1% penicillin-streptomycin (PEST, Sigma-Aldrich, MO), according to the manufacturer’s instructions. For all *in vitro* experiments, primary foetal or adult human cardiac fibroblasts were harvested and 4 x 10^5^ cells were seeded on six-well plates and cultured for seven days to confluency. Then, the cell culture medium was removed and cells were treated with DMSO or 10nM recombinant ADAMTS4 (4307, R&D Systems, MN) alone or in combination with 26nM ADAMTS inhibitor in fibroblast basal medium (115-500, Cell Applications Inc, CA) and cultured for an additional 24 hours. The concentration of 26nM of ADAMTS inhibitor was selected as it was the reported IC_50_ for proteoglycan cleavage,^1^ while an enzyme concentration of 10nM has been applied in previous studies.^9^ The conditioned cell culture medium was collected. Cells were lysed with buffer containing 1% SDS and 31.5mM Tris-HCl of pH 6.8.

**Concentration-response assessment of ADAMTS activity in human cardiac fibroblast lysates**

Primary foetal or adult human cardiac fibroblasts were cultured for seven days to confluency and cells were lysed with buffer containing 1% SDS and 31.5mM Tris-HCl, pH 6.8. Lysates were acetone precipitated and solubilised in assay buffer (50mM HEPES pH 7.4, 50mM NaCl and 5mM CaCl_2_). Protein concentration was measured and 200μg of lysates were incubated with DMSO or 1μg of recombinant ADAMTS4 or 11.2ng of ADAMTS inhibitor in a final volume of 100μl of assay buffer for 24 hours at 37°C on a shaker. In the concentration response experiments, lysates were treated with ADAMTS4, ADAMTS1 (2197, R&D Systems, MA) or ADAMTS5 (2198, R&D Systems, MA) at concentrations at 0.3125, 0.625, 1.25, 2.5, and 5 µg/100μl of lysate (highest concentration corresponds to 926 (ADAMTS1), 943 (ADAMTS4), and 1220 (ADAMTS5) nM).

**SDS-PAGE and western blotting for human cardiac fibroblast cultures**

To blot for ECM proteins, primary foetal or adult human cardiac fibroblasts were lysed with buffer that contained 1% SDS and 31.5mM Tris-HCl at pH 6.8. To blot for proteins in the conditioned medium, protein samples from cell culture supernatants were 10x up-concentrated using 3K Amicon centrifugal filters and diluted in 4x Laemmeli buffer. Protein samples were separated by electrophoresis using 4-15% SDS-polyacrylamide precast gels (BioRad, CA) under reducing (fibronectin) or non-reducing (LTBP1) conditions. Then, the purified proteins were transferred to a nitrocellulose or PVDF membrane using the Trans-Blot Turbo system (BioRad, CA). Thirty micrograms of cell lysates or 30μl of conditioned media were loaded per well of 4-15% TGX gel. Membranes were analysed with the following antibodies: rabbit anti-DPEAAE versican immunoglobulin G (IgG) (PA1-1748A, Invitrogen, MA), mouse anti-EDA-fibronectin IgG (clone IST9, Abcam, UK), mouse anti-EDA-fibronectin IgG (F6140, Sigma-Aldrich, MO), rabbit anti-fibronectin IgG (ab2413, Abcam, UK), rabbit anti-fibronectin IgG (15613-1-AP, Proteintech, IL), mouse anti-LTBP1 IgG (ab388, R&D Systems, MN), rabbit anti-LTBP1 IgG (268855, Proteintech, IL), and mouse anti-vinculin IgG (V9131, Sigma-Aldrich, MO). Blots were developed using the ECL system (Pierce, ThermoFisher Scientific, MA) and were imaged using ImageQuant™ LAS 4000 (GE Healthcare, IL) or Azure c600 (Azure Biosystems, CA). For some experiments, membranes were stained with Revert™ Total Protein Stain (Li-Cor, NE) and were imaged using Azure c600.

**Immunohistochemistry of human cardiac fibroblast cultures**

Primary foetal or adult human cardiac fibroblasts were harvested and 10^5^ cells were seeded on 24-well plates that contained coverslips (12mm diameter, 1.5H, Marienfeld, Germany) and cultured for seven days to confluency. The cell culture medium was removed and cells were treated with DMSO or 10nM recombinant ADAMTS4 alone or in combination with 26nM ADAMTS inhibitor in fibroblast basal medium (115-500, Cell Applications Inc, CA) and cultured for an additional 24 hours. Then, cells were fixed with 4% paraformaldehyde/PBS for 10 minutes at room temperature. After washing with PBS, cells were permeabilised in 0.5% Triton X-100/PBS buffer for 10 minutes and blocked with blocking buffer of 5% bovine serum albumin/PBS with 0.1% Triton X-100 for 1 hour at room temperature. Next, coverslips were incubated with primary antibody in blocking buffer for 1 hour at 37°C. Cells were then washed with 0.05% Tween-20/PBS and incubated with secondary antibody conjugated with Alexa fluor 488 goat IgG, Alexa fluor 546 goat IgG, or Alexa fluor 647 goat IgG (1:400, Invitrogen, MA) for 1 hour at room temperature. After washing, coverslips were mounted with VECTASHIELD vibrance mounting medium with DAPI (H-1800, Vector Laboratories, UK). Cells were visualised and pictures were acquired with a confocal laser scanning microscope (LSM 800, Zeiss, Germany). Fibronectin- and LTBP1-positive stained areas were quantified in an automated fashion using Cell Profiler image analysis software (www.cellprofiler.org).^10^ Confocal Z-stack images (n=8-9 representative images per experiment) from three independent experiments were quantified through the use of a pipeline that was designed to measure the area of the nucleus (DAPI staining), fibronectin and LTBP1. Area measurements of fibronectin and LTBP1 were quantified relative to the cell numbers (DAPI area) and normalised to DMSO controls. The following primary antibodies were used for immunocytofluorescence experiments: mouse anti-EDA-fibronectin IgG (F1640, Sigma-Aldrich, MO), rabbit anti-fibronectin IgG (ab2413, Abcam, UK) or mouse anti-LTBP1 IgG (mAb388, R&D Systems, MN).

**TGF-β quantification by tMLC**

To quantify total TGF-β in the conditioned media, we used tMLC cells as previously described,^11^ which were generously provided by Daniel Rifkin at New York University School of Medicine. In 48-well plates, 4x10^5^ tMLC cells were cultured in serum-free conditions for 120 minutes. Adult human cardiac fibroblasts were harvested and 4x10^5^ cells were seeded on six-well plates and cultured for seven days to confluency. Then, the cell culture medium was removed and cells were treated with DMSO or 10nM recombinant ADAMTS4 alone or in combination with 26nM ADAMTS inhibitor in fibroblast basal medium (115-500, Cell Applications Inc, CA) and cultured for an additional 24 hours. The conditioned cell culture medium was collected and heated for 10 minutes at 80^o^C to activate latent TGF-β. Recombinant TGF-β or heat-activated, conditioned media were diluted 1:1 in serum-free Dulbecco’s modified eagle medium (200µl media and 200µl conditioned media) and added to tMLC cultures in triplicate. After 16 hours, cells were lysed and luciferase activity was assessed by treating the lysates with luciferin (Promega, WI). Luminescence was measured using a Hidex Sense microplate reader.

**Cleavage of recombinant peptides**

For experiments on fibronectin digestion by ADAMTS4, 1μg of recombinant fibronectin fragments (R&D Systems, MN) was used as substrates for 0.5μg recombinant ADAMTS4. We digested recombinant C-terminal HIS-tagged fibronectin peptides covering FIII-1 to FIII-7 (mid-region peptide, 3225-FN) and FIII-8 to FIII-12 including the extradomains A and B (ED-containing peptide, 3938-FN) with ADAMTS4. Fragments were separated by SDS-PAGE and visualised by total protein staining.

**Supplementary Results**

**Causes of death and adverse effects**

We assessed causes of death based on observations before death and necropsy. Causes of death included advanced heart failure, intrathoracic bleeding, the need to kill the animals due to wound rupture and intolerance to isoflurane anaesthesia during MRI examination, and were assessed based on necropsy and observations pre-mortem (Table S4). The rats were considered to have decompensated heart failure if we found an increased lung/body weight ratio (>10mg/g) or signs of lung congestion by visual inspection at necropsy (n=6). Rats that were found dead without apparent reason and lacking necropsy were also considered to have died of heart failure (n=6). The deaths from suspected heart failure were different among the groups (ADAMTS inhibitor n=3 (13%) vs. vehicle n=9 (38%), HR 0.26 (0.07-0.95), p=0.041, log-rank p=0.027) (Figure S2). Intrathoracic bleeding is a known complication of AB^12^, and tended to be more common among rats treated with vehicle than among those treated with ADAMTS inhibitor (vehicle n=5, ADAMTS inhibitor n=1, p=0.188). In contrast, we observed a tendency towards a higher prevalence of wound ruptures and revisions in AB rats treated with ADAMTS inhibitor than in those treated with vehicle (vehicle n=4 vs. ADAMTS inhibitor n=11, p=0.060). Three AB rats (vehicle n=2, ADAMTS inhibitor n=1, p=1.000) died during isoflurane anaesthesia during MRI examinations.

**Deviations from study protocol, *in vivo* study**

One AB rat in the vehicle-treated group that died due to intrathoracic bleeding, received one dose of ADAMTS inhibitor. One sample taken from a vehicle-treated AB rat was lost before laboratory analysis could be performed. This rat had a heart function similar to the mean of this group, and the loss of this sample is not expected to have influenced the results substantially except in the loss in power. One outlier was removed in the sham vehicle group from RT-qPCR due to values twice the standard deviation from the mean of the group for several genes measured.

**Cleavage of fibronectin**

Cleavage activity of ADAMTS4 towards the EDA-fibronectin was determined by incubating ADAMTS4 with a peptide that contained the EDA. Incubation of this peptide with ADAMTS4 eliminated much of the full-length peptide, and generated a 12kDa His-tagged fragment, indicating a cleavage site around aa1773-1818 that corresponded to the EDA domain. A control peptide of the mid-region of fibronectin was not cleaved (Figure S3).

**Validation of enzyme activity**

To confirm that the recombinant ADAMTS4 possessed known cleavage activities, the amounts of versican DPEAAE fragments, specific for ADAMTS4-mediated cleavage of versican^1^, were quantified in our *in vitro* system. The amounts of DPEAAE fragments were markedly increased upon treatment with ADAMTS4 and were prevented by the inhibitor in human cardiac fibroblast lysates and cultures (Figure S5).

**Supplementary Tables**

**Supplementary Table 1. Echocardiography before randomisation (day 1)**

|  | AB vehicle | AB inhibitor | *p* |
| --- | --- | --- | --- |
| n | 24 | 24 |  |
| Body weight (g) | 288 ± 33 | 289 ± 36 | *0.898* |
| Gradient over banding (m/s) | 4.27 ± 0.85 | 4.31 ± 0.89 | *0.882* |
| Left atrial diameter (mm) | 4.21 ± 0.72 | 4.20 ± 0.77 | *0.939* |
| Left ventricular diameter in diastole (mm) | 7.45 ± 0.54 | 7.58 ± 0.50 | *0.406* |

AB, aortic banding; inhibitor, ADAMTS inhibitor. Groups are compared by Student *t*-test.

**Supplementary Table 2. Dose simulation of oral administration of ADAMTS** **inhibitor in rats**

| Oral o.d. dose (mg/kg) | Css, total (μM) | Css, unbound (μM) | Css, unbound/IC_50_ |
| --- | --- | --- | --- |
| 10 | 0.36 | 0.18 | 7.09 |
| 15 | 0.54 | 0.27 | 10.6 |
| 20 | 0.72 | 0.36 | 14.2 |

o.d., once daily; Css, concentration in steady state.

**Supplementary Table 3. Cardiac function and structure**

|  | Sham vehicle | Sham inhibitor | p (sham: vehicle vs. inhibitor) | AB vehicle | p (vehicle: sham  vs. AB) | AB inhibitor | p (AB: vehicle vs. inhibitor) |
| --- | --- | --- | --- | --- | --- | --- | --- |
| n | 6 | 6 |  | 8 |  | 17 |  |
| Organ weights |  |  |  |  |  |  |  |
| BW (g) | 459 ± 40 | 467 ± 36 | *1.000* | 444 ± 33 | *1.000* | 441 ± 41 | *1.000* |
| LV/BW (mg/g) | 1.88 ± 0.14 | 1.83 ± 0.12 | *1.000* | 3.09 ± 0.41 | ***<0.001*** | 2.63 ± 0.39 | ***0.009*** |
| RV/BW (mg/g) | 0.50 ± 0.07 | 0.49 ± 0.09 | *1.000* | 0.52 ± 0.08 | *1.000* | 0.51 ± 0.09 | *1.000* |
| Lung/BW (mg/g) | 3.50 ± 0.56 | 3.28 ± 0.44 | *1.000* | 3.95 ± 0.72 | *0.476* | 3.61 ± 0.57 | *0.553* |
| Echocardiography |  |  |  |  |  |  |  |
| IVDd (mm) | 1.61 ± 0.14 | 1.61 ± 0.09 | *1.000* | 2.16 ± 0.15 | ***<0.001*** | 1.89 ± 0.14 | ***<0.001*** |
| PWDd (mm) | 1.58 ± 0.12 | 1.64 ± 0.05 | *1.000* | 2.16 ± 0.12 | ***<0.001*** | 1.84 ± 0.16 | ***<0.001*** |
| LVDd (mm) | 8.21 ± 0.51 | 7.72 ± 0.54 | *0.475* | 8.53 ± 0.84 | *0.996* | 7.76 ± 0.48 | ***0.014*** |
| LVDs (mm) | 4.48 ± 0.58 | 3.96 ± 0.29 | *0.647* | 5.31 ± 1.09 | *0.120* | 3.90 ± 0.64 | ***<0.001*** |
| FS (%) | 46 ± 5 | 49 ± 4 | *1.000* | 38 ± 9 | *0.166* | 50 ± 7 | ***0.001*** |
| LAD (mm) | 3.86 ± 0.18 | 3.74 ± 0.44 | *1.000* | 5.57 ± 1.22 | ***0.016*** | 4.03 ± 0.37 | ***0.027*** |
| LVOT/aorta (mm) | 2.54 ± 0.19 | 2.67 ± 0.07 | *0.462* | 2.69 ± 0.14 | *0.219* | 2.60 ± 0.16 | *0.438* |
| E (mm/s) | 945 ± 109 | 994 ± 91 | *1.000* | 1092 ± 203^*^ | *0.192* | 1000 ± 127 | *0.443* |
| e’ (mm/s) | 57.4 ± 10.9 | 54.2 ± 4.6 | *1.000* | 42.7 ± 6.6 | ***0.010*** | 55.8 ± 9.5^*^ | ***0.004*** |
| E/e’ | 16.8 ± 2.4 | 18.5 ± 2.6 | *1.000* | 25.9 ± 5.9^*^ | ***0.001*** | 18.4 ± 4.0^*^ | ***0.001*** |
| Systolic tissue velocity (cm/s) | 60.0 ± 6.5 | 56.5 ± 7.8 | *1.000* | 35.2 ± 6.7 | ***<0.001*** | 46.7 ± 6.1^*^ | ***0.001*** |
| HR (/s) | 369 ± 19 | 394 ± 26 | *0.611* | 314 ± 39 | ***0.011*** | 352 ± 35 | ***0.032*** |
| Magnetic resonance imaging |  |  |  |  |  |  |  |
| *Cine* |  |  |  |  |  |  |  |
| LV volume diastole (mm^3^) | 673 ± 67^*^ | 613 ± 60 | 0.811 | 693 ± 115 | *1.000* | 624 ± 86 | *0.228* |
| LV volume systole (mm^3^) | 233 ± 82^1^ | 205 ± 33 | *1.000* | 331 ± 98 | *0.146* | 224 ± 89 | ***0.017*** |
| EF (%) | 66 ± 9^*^ | 66 ± 6 | *1.000* | 53 ± 7 | *0.076* | 65 ± 11 | ***0.021*** |
| HR (/min) | 335 ± 31 | 351 ± 45 | *1.000* | 320 ± 39 | *1.000* | 321 ± 33 | *1.000* |
| *Strain* |  |  |  |  |  |  |  |
| LV maximal longitudinal (%) | -14.6 ± 4.3^†^ | -16.2 ± 1.5 | *1.000* | -9.0 ± 3.8 ^*^ | ***0.043*** | -12.5 ± 3.5^†^ | *0.104* |

Data presented in mean ± standard deviation. Groups were compared by one-way ANOVA with planned comparisons followed by Bonferroni correction for the following comparisons: sham vehicle vs. sham ADAMTS inhibitor, sham vehicle vs. AB vehicle, AB vehicle vs. AB ADAMTS inhibitor. P-values <0.05 were considered significant and marked in bold.

Inhibitor, ADAMTS inhibitor; BW, body weight; LV, left ventricle; RV, right ventricle; IVSd, interventricular septum in diastole; PWDd, posterior wall diameter in diastole; LVDd, left ventricular diameter in diastole; LVDs, left ventricular diameter in systole; FS, fractional shortening; LAD, left atrial diameter; LVOT, left ventricular outflow tract; E, peak mitral inflow velocity; e’, peak diastolic tissue velocity; EF, ejection fraction; HR, heart rate. ^*^Missing one animal; ^†^ missing two animals.

**Supplementary Table 4. Causes of death**

| Group | Days after AB | Cause of death | Evaluation based on |
| --- | --- | --- | --- |
| AB vehicle | 4 | Intrathoracic bleeding | Necropsy |
| AB vehicle | 4 | Heart failure | Necropsy |
| AB vehicle | 4 | Heart failure | Necropsy |
| AB vehicle | 4 | Intrathoracic bleeding | Necropsy |
| AB inhibitor | 5 | Intrathoracic bleeding | Necropsy |
| AB inhibitor | 5 | Heart failure | Necropsy |
| AB inhibitor | 5 | Unknown | Not examined |
| AB vehicle | 5 | Intrathoracic bleeding | Necropsy |
| AB vehicle | 6 | Intrathoracic bleeding | Necropsy |
| AB vehicle | 6 | Unknown | Not examined |
| AB vehicle | 6 | Unknown | Not examined |
| AB vehicle | 9 | Intrathoracic bleeding | Necropsy |
| AB vehicle | 9 | Heart failure, sacrificed | Observation |
| AB vehicle | 21 | Heart failure, sacrificed | Necropsy |
| AB inhibitor | 25 | Unknown | Not examined |
| AB vehicle | 34 | Heart failure | Necropsy |
| AB inhibitor | 43 | Wound rupture, sacrificed | Observation |
| AB vehicle | 44 | Unknown | Not examined |
| AB inhibitor | 46 | Wound rupture, sacrificed | Observation |
| AB vehicle | 51 | Unknown | Not examined |
| AB inhibitor | 52 | Isoflurane anaesthesia during MRI | Observation |
| AB vehicle | 52 | Isoflurane anaesthesia during MRI | Observation |
| AB vehicle | 53 | Isoflurane anaesthesia during MRI | Observation |

AB, aortic banding; ADAMTS4, a disintegrin and metalloprotease with thrombospondin motif; MRI, magnetic resonance imaging.

**Supplementary Table 5. The mRNA expression of genes encoding for ADAMTS proteoglycanases, hypertrophic and inflammatory regulators in pressure-overloaded rats**

|  | Sham vehicle | Sham inhibitor | p (sham:  vehicle vs inhibitor) | AB vehicle | p (vehicle:  sham vs AB) | AB inhibitor | p (AB:  vehicle vs inhibitor) |
| --- | --- | --- | --- | --- | --- | --- | --- |
| n | 5 | 6 |  | 7 |  | 17 |  |
| Adamts |  |  |  |  |  |  |  |
| Adamts1 | -0.03 ± 0.32 | -0.04 ± 0.64 | *1.000* | 0.32 ± 0.24 | *0.874* | 0.56 ± 0.63 | *0.960* |
| Adamts4 | -0.13 ± 0.65 | 0.02 ± 1.45 | *1.000* | 1.17 ± 0.61 | *0.181* | 0.59 ± 1.26 | *0.796* |
| Adamts5 | -0.03 ± 0.31 | -0.17 ± 0.42 | *1.000* | -0.19 ± 0.34 | *1.000* | 0.01 ± 0.49 | *0.983* |
| Adamts9 | -0.25 ± 0.98 | -0.14 ± 1.36 | *1.000* | -0.55 ± 2.61 | *1.000* | 0.37 ± 1.48 | *0.718* |
| Adamts15 | -0.26 ± 1.00 | -0.50 ± 0.93 | *1.000* | -0.12 ± 0.64 | *1.000* | -0.32 ± 0.93 | *1.000* |
| Adamts20 | -0.28 ± 1.02 | -0.01 ± 1.29 | *1.000* | -0.25 ± 1.03 | *1.000* | 0.45 ± 1.20 | *0.575* |
| Timp3 | -0.02 ± 0.30 | -0.10 ± 0.17 | *1.000* | 0.11 ± 0.25 | *1.000* | 0.05 ± 0.36 | *1.000* |
| Hypertrophy |  |  |  |  |  |  |  |
| Nppa | -0.24 ± 0.97 | -1.40 ± 1.43 | *0.695* | 3.18 ± 1.36 | ***0.002*** | 1.95 ± 1.77 | *0.268* |
| Nppb | -0.06 ± 0.49 | 0.23 ± 0.60 | *1.000* | 1.68 ± 0.40 | ***<0.001*** | 1.29 ± 0.65 | *0.442* |
| Acta1 | -0.47 ± 1.32 | -0.33 ± 1.35 | *1.000* | 1.39 ± 2.55 | *0.220* | 1.89 ± 1.49 | *1.000* |
| Myh6 | -0.15 ± 0.72 | 0.01 ± 1.34 | *1.000* | -1.09 ± 1.92 | *0.838* | -0.14 ± 1.43 | *0.464* |
| Myh7 | -0.24 ± 0.87 | -0.66 ± 0.87 | *1.000* | 0.86 ± 1.55 | *0.283* | 0.95 ± 0.97 | *1.000* |
| Inflammation |  |  |  |  |  |  |  |
| Tnf | -0.16 ± 0.82 | 1.13 ± 1.18 | *0.206* | 1.75 ± 1.19 | ***0.021*** | 0.86 ± 1.16 | *0.264* |
| Il1b | -0.18 ± 0.83 | 0.46 ± 0.81 | *1.000* | 1.35 ± 1.60 | *0.310* | 0.93 ± 1.84 | *1.000* |
| Il6 | -0.67 ± 1.55 | 0.51 ± 2.47 | *1.000* | 4.90 ± 3.01 | ***0.034*** | 4.01 ± 4.27 | *1.000* |
| Ccl2 | -0.14 ± 0.69 | 0.62 ± 0.55 | *0.140* | 0.81 ± 0.51 | ***0.036*** | 1.04 ± 0.64 | *1.000* |

Values are given in log2-transformed fold change expression to sham vehicle and normalized to RPL4. Groups were compared by one-way ANOVA with planned comparisons followed by Bonferroni correction for the following comparisons: sham vehicle vs. sham ADAMTS inhibitor, sham vehicle vs. AB vehicle, AB vehicle vs. AB ADAMTS inhibitor. P-values <0.05 were considered significant and marked in bold.

Inhibitor, ADAMTS inhibitor.

**Supplementary Table 6**

| Patient characteristics, explanted hearts (n=8) | |
| --- | --- |
| Female gender | 2 (25) |
| Age (years) | 42 ± 13 |
| BMI | 24 ± 2 |
| Current smoker | 1 (13) |
| Duration of heart failure (years) | 6 ± 5 |
| NYHA functional class IV | 4 (50) |
| Aetiology | |
| Unknown | 5 (63) |
| Previous viral myocarditis | 2 (25) |
| Muscular dystrophia | 1 (13) |
| Heart failure therapy | |
| Mechanical circulatory support | 3 (38) |
| CRT | 3 (38) |
| ACEi or ARB | 7 (88) |
| Betablocker | 5 (63) |
| MRA | 3 (38) |
| Clinical chemistry | |
| Haemoglobin (g/dl) | 13.4 ± 1.4 |
| Creatinine (µmol/l) | 99 ± 33 |
| NT-proBNP (pg/ml) | 564 ± 705 |
| Echocardiography |  |
| IVSd (cm) | 0.8 ± 0.2 |
| LVPWd (cm) | 0.7 ± 0.2 |
| LVIDd (cm) | 7.7 ± 0.9 |
| LVIDs (cm) | 7.2 ± 0.9 |
| CO (l/min) | 2.7 ± 0.9 |
| EF (%) | 17 ± 2 |
| E/A | 3.0 ± 1.3 |
| Right atrium (mmHg) | 7 ± 5 |
| PA mean (mmHg) | 23 ± 9 |
| PCWP (mmHg) | 14 ± 8 |
| RV CO (L/min) | 4.6 ± 1.0 |

Values are presented in number (%) unless otherwise specified. BMI, body mass index; NYHA, New York Heart Association; mechanical circulatory support includes intra-aortic balloon pump, extracorporeal membrane oxygenation, and LV assist device; CRT, cardiac resynchronisation therapy; ACEi, ACE-inhibitors, ARB, angiotensin II receptor blockers; MRA, mineralocorticoid receptor antagonist; NT-proBNP, N-terminal pro-brain natriuretic peptide; IVSd, interventricular septum thickness in diastole; LVPWd, LV posterior wall thickness in diastole; LVIDd, LV internal diameter in diastole; LVIDs, LV internal diameter in systole; CO, cardiac output; EF, ejection fraction; E/A, E-wave to A-wave ratio; PA, pulmonary artery; PCWP, pulmonary capillary wedge pressure, RV, right ventricle.

**Supplementary Table 7. Characteristics of 6 weeks AB rats**

|  | Sham | AB | p (AB vs sham) |
| --- | --- | --- | --- |
| n | 8 | 8 |  |
| BW (g) | 452 ± 40 | 356 ± 27 | ***<0.001*** |
| IVSd (mm) | 1.56 ± 0.19 | 1.97 ± 0.17 | ***<0.001*** |
| PWDd (mm) | 1.61 ± 0.11 | 2.07 ± 0.13 | ***<0.001*** |
| LVDd (mm) | 8.15 ± 0.40 | 8.72 ± 0.60 | ***0.043*** |
| LVDs (mm) | 4.84 ± 0.62 | 6.32 ± 0.79 | ***0.001*** |
| FS (%) | 41 ± 6 | 28 ± 5 | ***<0.001*** |
| LAD (mm) | 3.57 ± 0.18 | 6.21 ± 0.53 | ***<0.001*** |
| E (mm/s) | 858 ± 122 | 1415 ± 163 | ***<0.001*** |
| e’ (mm/s) | 51 ± 10 | 53 ± 11 | *0.748* |
| E/e’ | 17.0 ± 2.7 | 27.3 ± 4.6 | ***<0.001*** |
| HR (/s) | 375 ± 36 | 328 ± 29 | ***0.012*** |

Data presented in mean ± standard deviation. Groups were compared by student *t*-test. P-values <0.05 were considered significant and marked in bold.

AB, aortic banding; BW, body weight; LV, left ventricle; IVSd, interventricular septum in diastole; PWDd, posterior wall diameter in diastole; LVDd, left ventricular diameter in diastole; LVDs, left ventricular diameter in systole; FS, fractional shortening; LAD, left atrial diameter; E, peak mitral inflow velocity; e’, peak diastolic tissue velocity; HR, heart rate.

**Supplementary Figures**


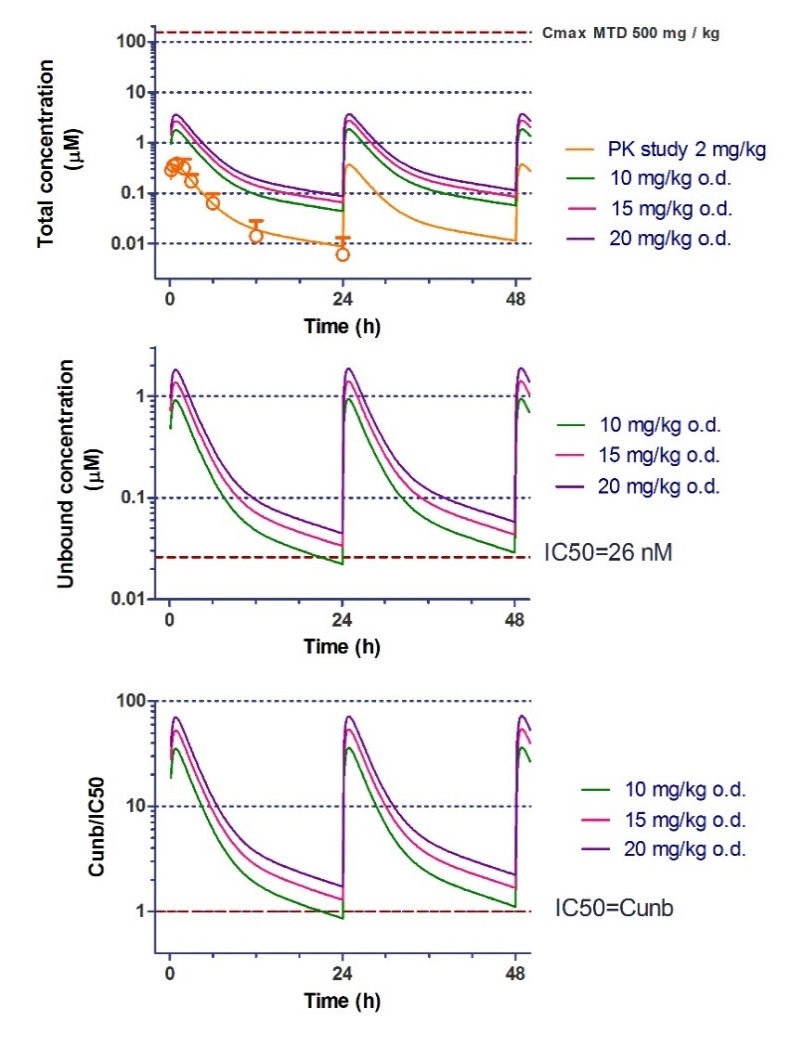
**Supplementary Figure 1. Dose simulations for oral administration of ADAMTS inhibitor in rats.** Dose simulations for oral administration of ADAMTS inhibitor based on Cmax and T_1/2_ in a pharmacokinetic study for 2mg/kg oral administration. Cmax, maximal plasma concentration; MTD, maximum tolerated dose; Cunb, unbound concentration.


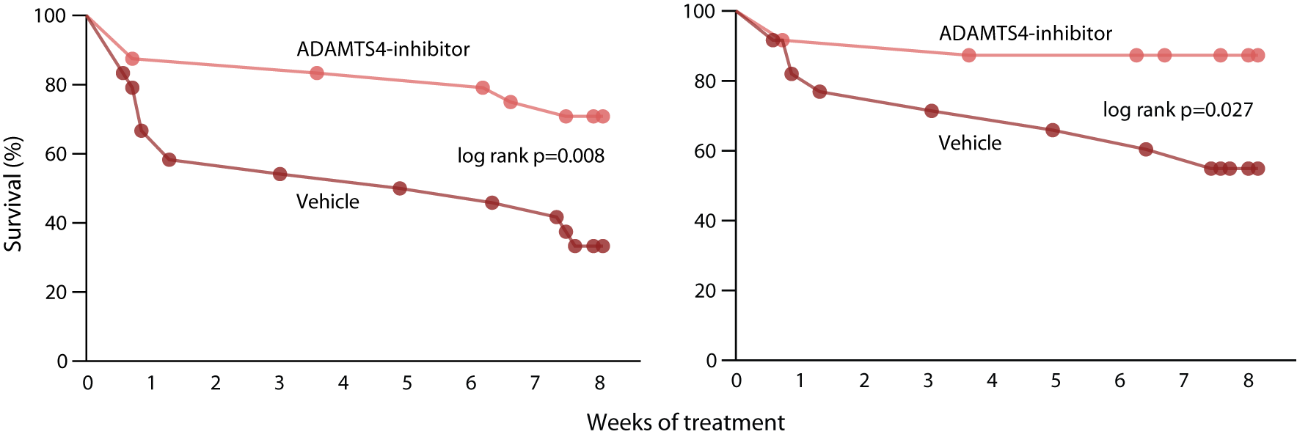


**Supplementary Figure 2. Cardiac mortality.** Kaplan Meier plot for total mortality (left) and mortality suspected to be related to heart failure (right). Differences in mortality rates between AB rats treated with ADAMTS inhibitor (pink) and those treated with vehicle (red) calculated by log-rank test.

AB, aortic banding; ADAMTS4, a disintegrin and metalloprotease with thrombospondin motif.


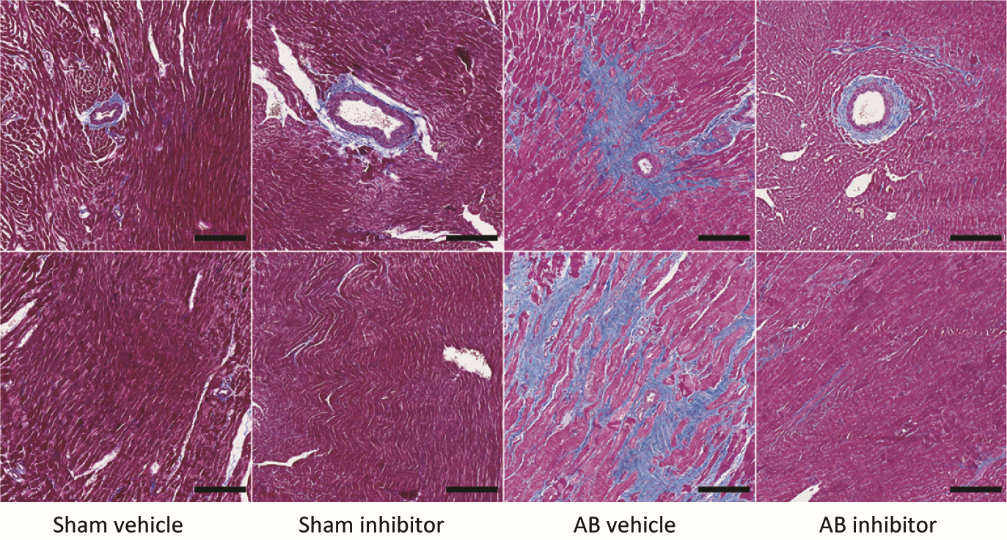


**Supplementary Figure 3. Localisation of fibrosis.** Representative images of myocardial sections stained with Masson’s trichrome, where the top panel represents areas of perivascular fibrosis and lower panel represents interstitial fibrosis. Scale bars represent 20 microns.


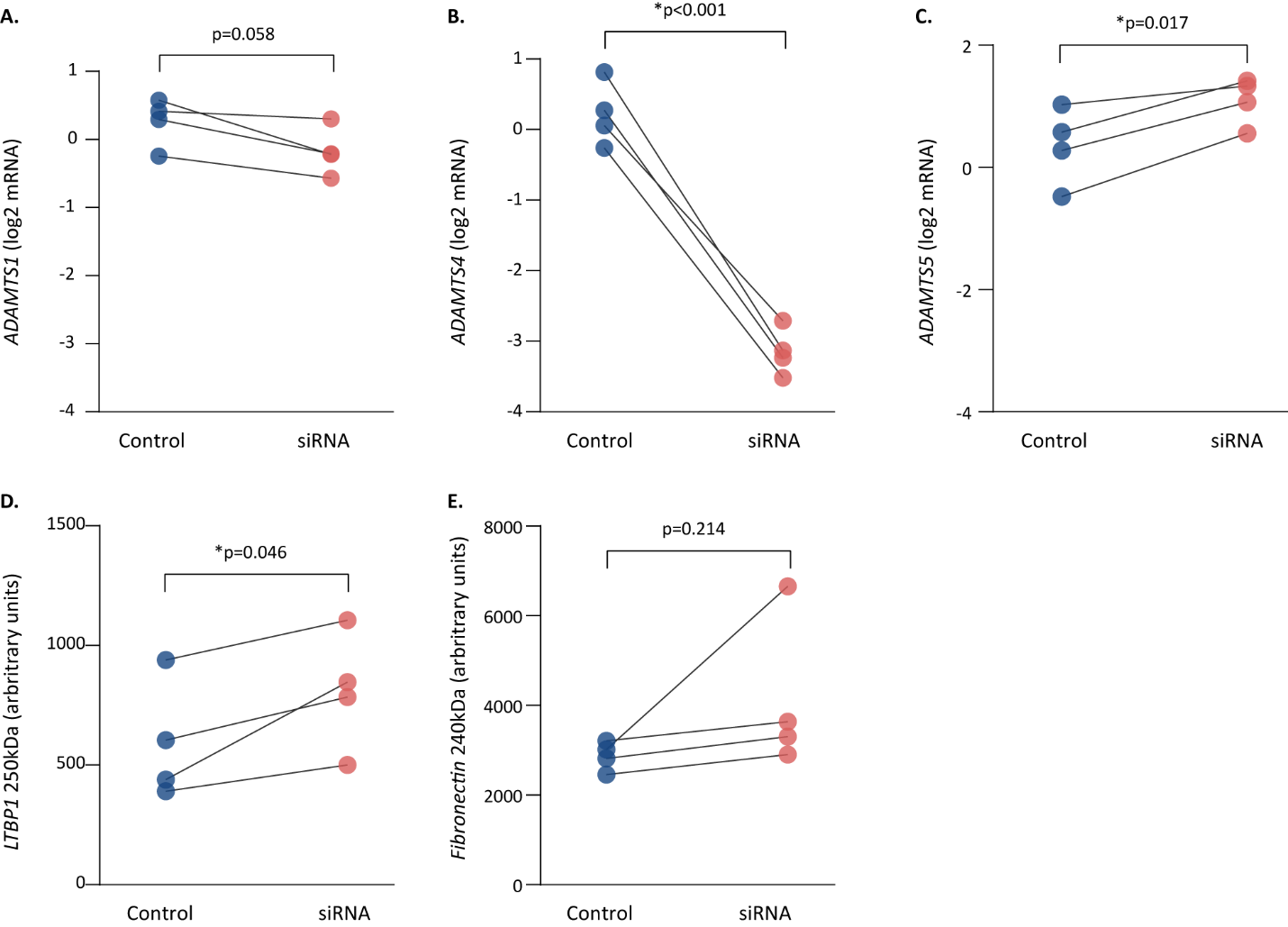


**Supplementary Figure 4. Effects of gene knockdown of ADAMTS4 with siRNA.** (**A-C**) log2 transformed mRNA levels of ADAMTS1 (A), ADAMTS4 (B), and ADAMTS5 (C) and (**D-E**) protein levels of LTBP1 (D) and fibronectin (E) in cultures of human cardiac fibroblasts treated with siRNA for ADAMTS4.

P-values <0.05 were considered significant and are marked with *.


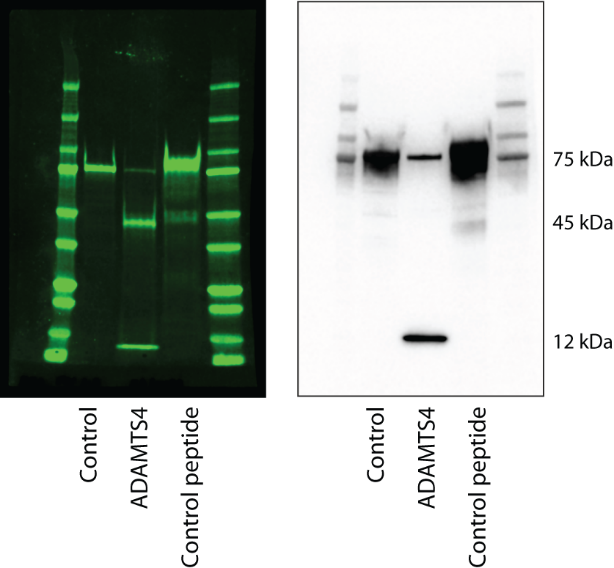


**Supplementary Figure 5.** Cleavage of EDA-containing fibronectin peptide (full-length 75kDa) demonstrates a C-terminal His-tagged fragment of 12kDa, and a non-C-terminal fragment of 45 kDa. Blots show His-staining (right) and total protein stain (left), of the following samples: Control treatment of EDA-containing peptide (Control), ADAMTS4 incubated with EDA-containing peptide (ADAMTS4) and ADAMTS4 incubated with a mid-region fibronectin peptide (Control peptide).

**
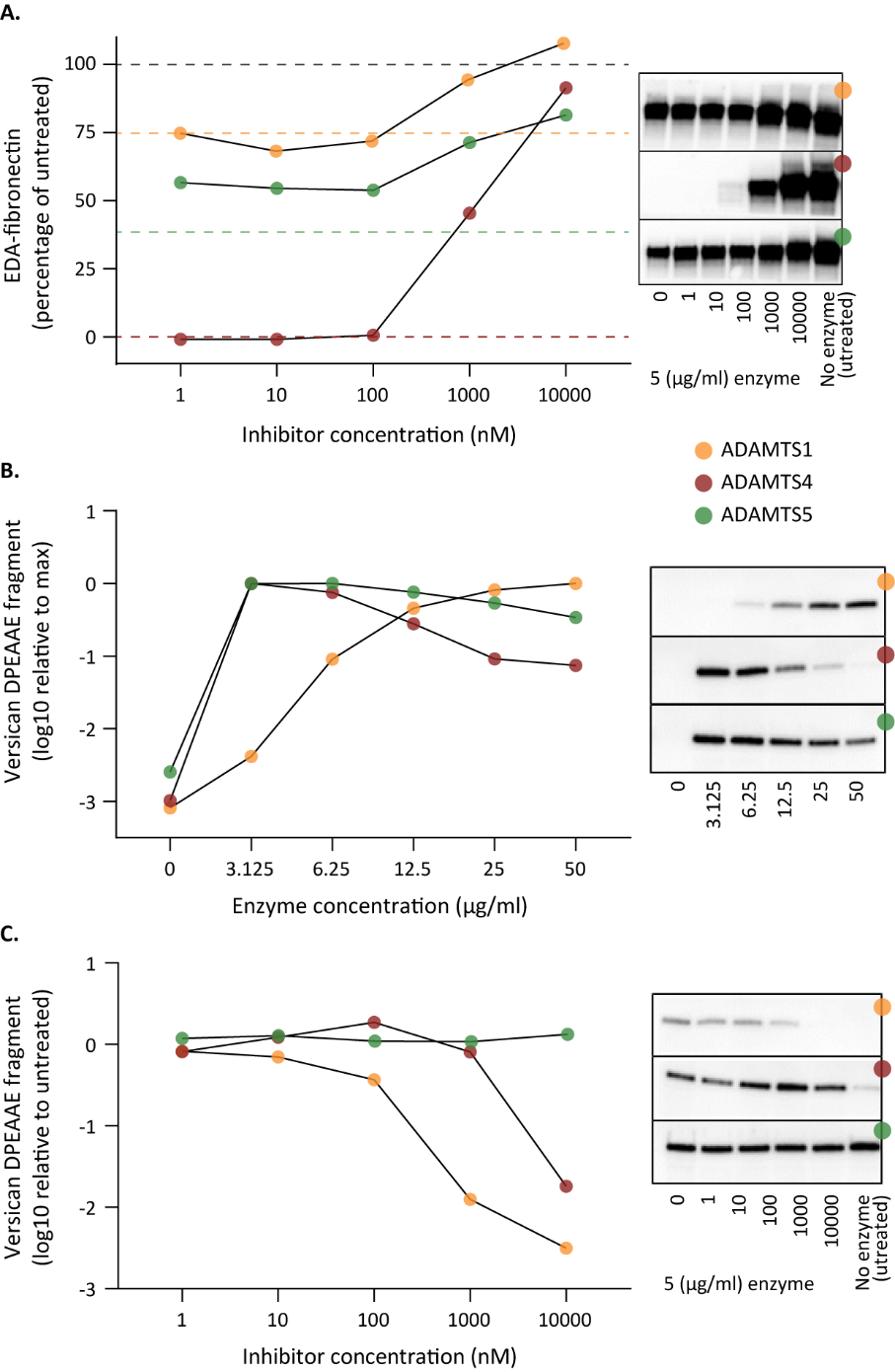
**

**Supplementary Figure 6**. (**A**) Inhibition of EDA-fibronectin cleavage by the ADAMTS inhibitor for increasing doses of ADAMTS1, -4 and -5. EDA-fibronectin cleavage determined as percentage of untreated sample of 240kDa EDA-specific band on immunoblots (representative images to right). Dotted lines indicate levels when no inhibitor is present. (**B**) Versican cleavage determined by the presence of DPEAAE-immunoreactive 70kDa fragments (representative immunoblots to right) in response to increasing concentrations of ADAMTS enzymes. (**C**) Inhibition of versican cleavage by ADAMTS enzymes in increasing doses of the ADAMTS inhibitor.


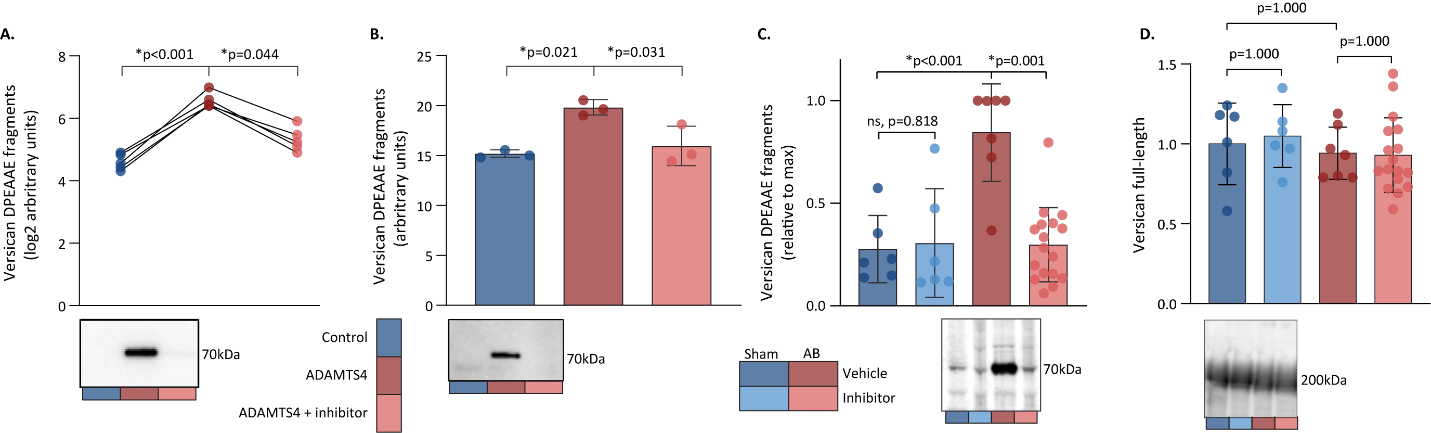


**Supplementary Figure 7.** (**A**) Release of versican fragments in cultures of foetal human cardiac fibroblasts treated with ADAMTS4 alone or in combination with ADAMTS inhibitor. Groups were compared by ANOVA for related samples. (**B**) Production of versican fragments in foetal human cardiac fibroblasts treated with ADAMTS4 alone or in combination with ADAMTS inhibitor. Groups were compared by one-way ANOVA with planned comparisons. (**C**) Amount of versican DPEAAE fragments in the myocardium of rats placed in the following groups: sham vehicle (n=6), sham ADAMTS inhibitor (n=6), AB vehicle (n=7), AB ADAMTS inhibitor (n=17). Bars represent means, while error bars indicate standard deviations. (**D**) Amount of 200kDa versican in myocardium of rats in the same groups as in (C).

Groups were compared by one-way ANOVA with planned comparisons followed by Bonferroni correction for the following comparisons: control vs. ADAMTS4 and ADAMTS4 vs. ADAMTS4 + inhibitor (A and B), and sham vehicle vs. sham ADAMTS4-inhibitor, sham vehicle vs. AB vehicle, AB vehicle vs. AB ADAMTS inhibitor (C). P-values <0.05 were considered significant and are marked with *. Representative blots are shown.


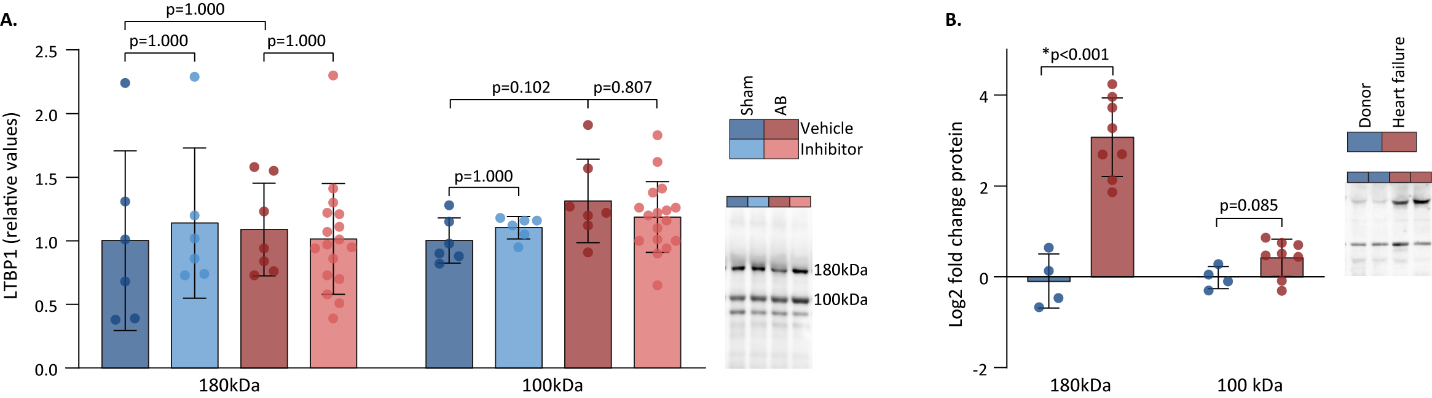


**Supplementary Figure 8. LTBP1 in vivo.** (**A**) The amount of LTBP1 full-length (180kDa) and fragment (100kDa) in the myocardium of rats placed in the following groups: sham vehicle (n=6), sham ADAMTS inhibitor (n=6), AB vehicle (n=7), AB ADAMTS inhibitor (n=17). Bars represent means, while error bars indicate standard deviations. Groups were compared by one-way ANOVA with planned comparisons followed by Bonferroni correction for the following comparisons: sham vehicle vs. sham ADAMTS inhibitor, sham vehicle vs. AB vehicle, AB vehicle vs. AB ADAMTS inhibitor. (**B**) The amount of LTBP1 in the myocardium of human hearts from healthy donors and heart failure patients.

P-values <0.05 were considered significant and are marked with *. Representative blots are shown.

**Echocardiographic images from Figure 1**

Sham vehicle

**
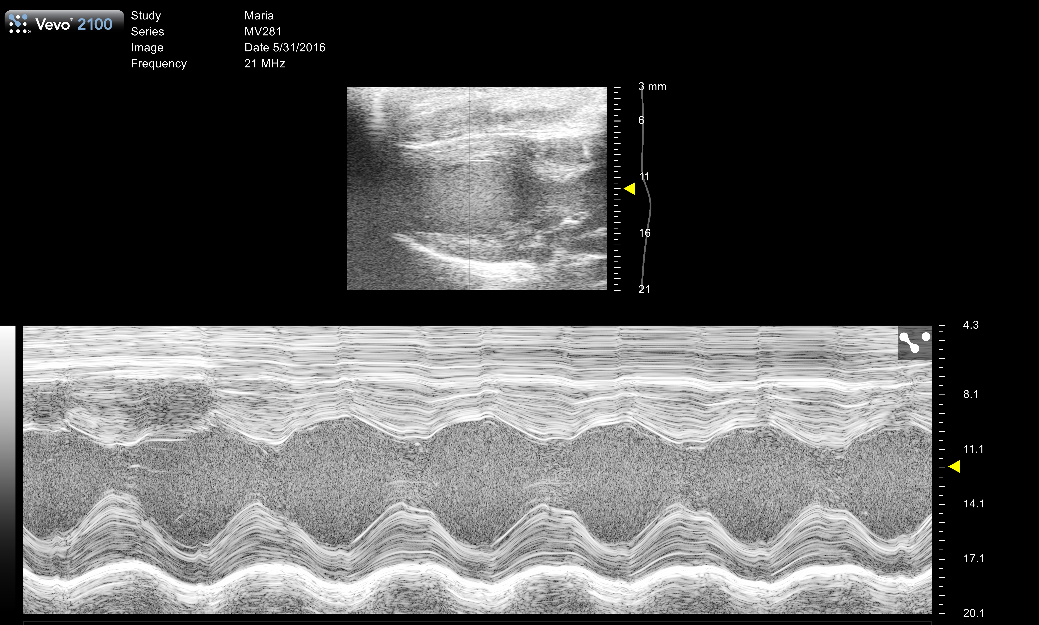
**

100 ms

Sham ADAMTS inhibitor

**
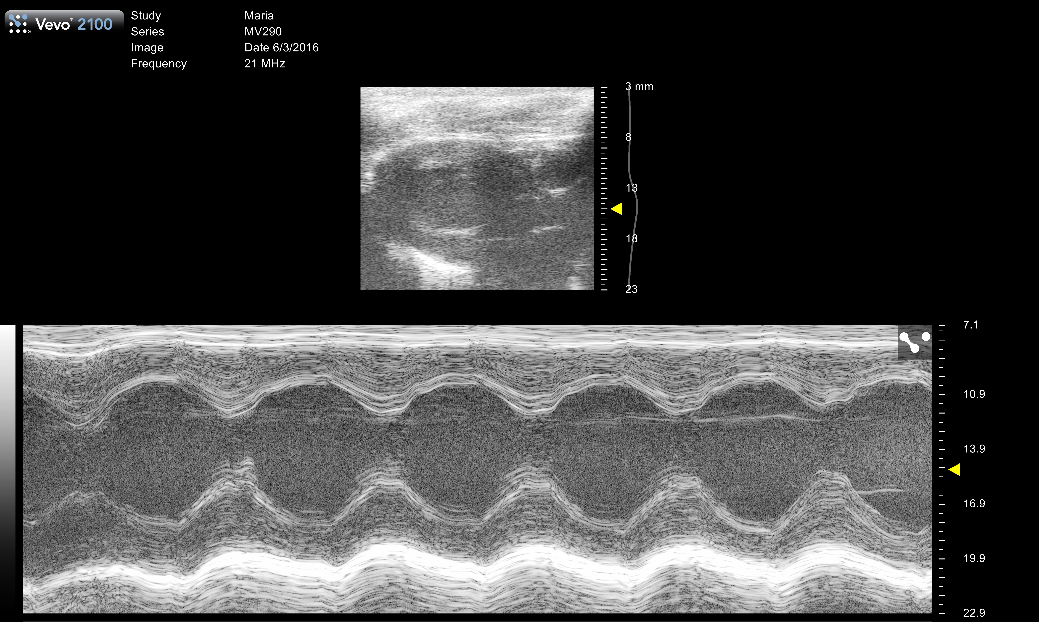
**

100 ms

Aortic banding vehicle

**
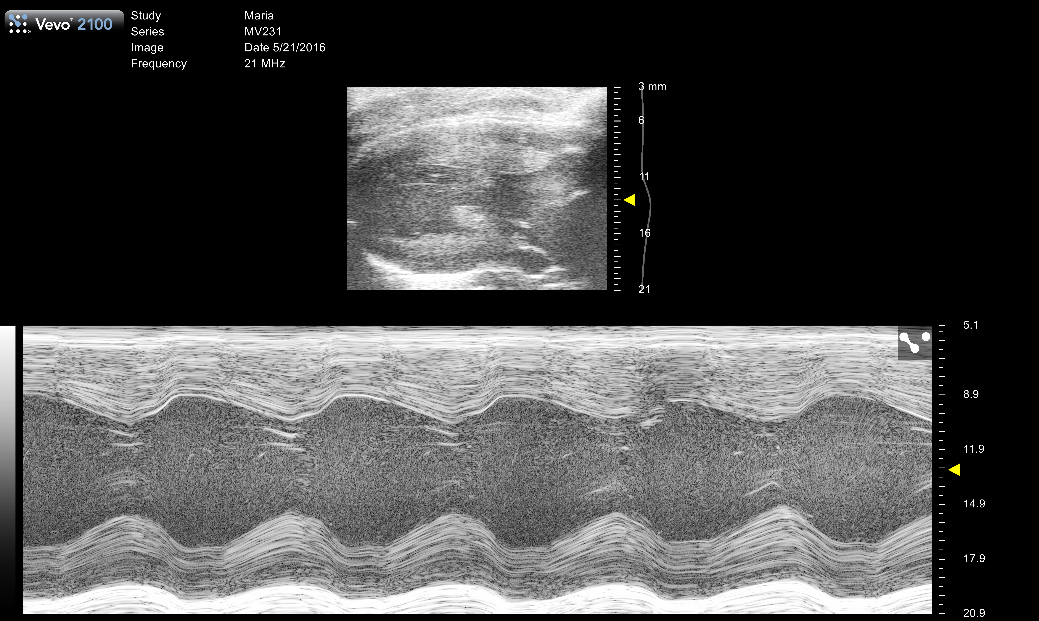
**

100 ms

Aortic banding ADAMTS inhibitor

**
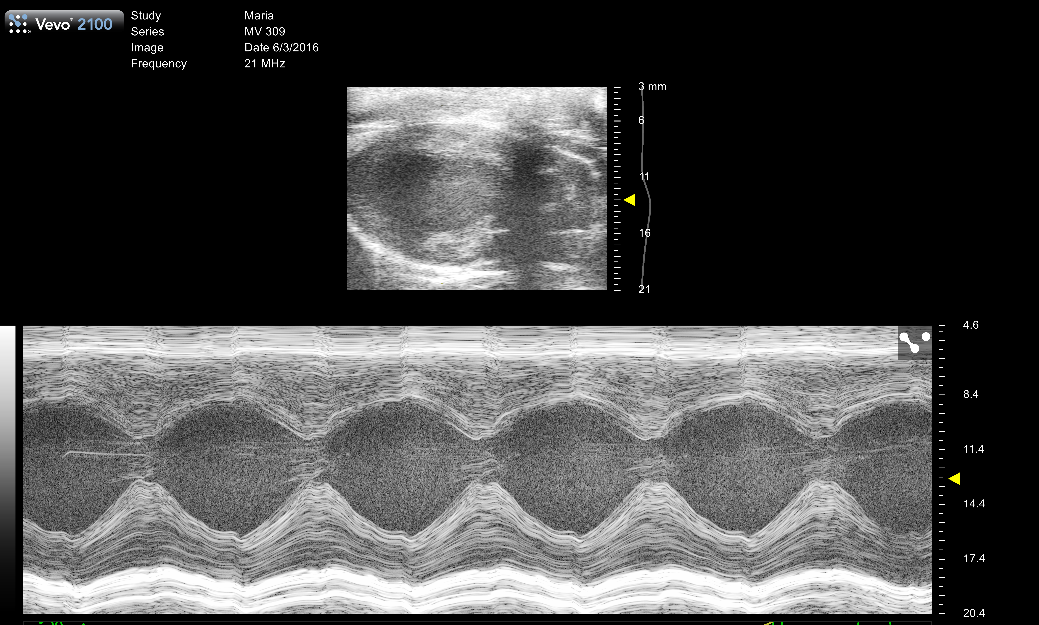
**

100 ms

**Complete unedited gels for each representative cropped gel**

*Lanes that correspond with the lanes shown in the cropped gels in the manuscript are marked with orange boxes. Loading controls (total protein stain or vinculin) are shown on the right side.*

**Blots from Figure 3E**


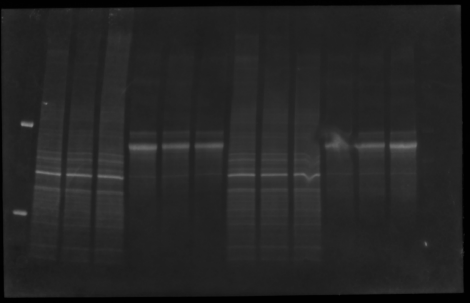

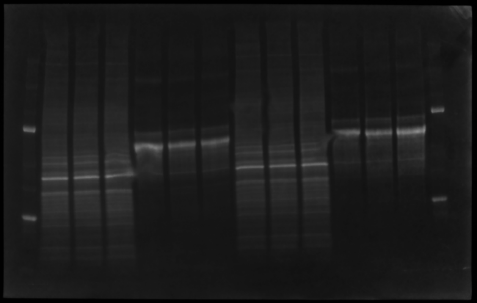
Full-length EDA-fibronectin in ECM
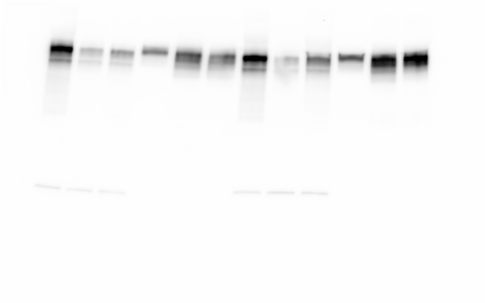


180-kDa fibronectin fragment in medium
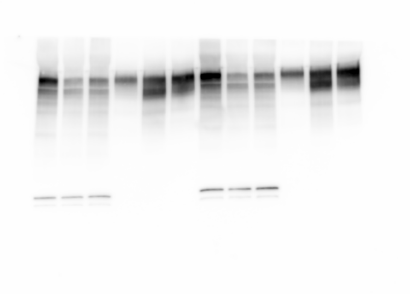


**Blots from Figure 3F**

Full-length LTBP1 in ECM

**
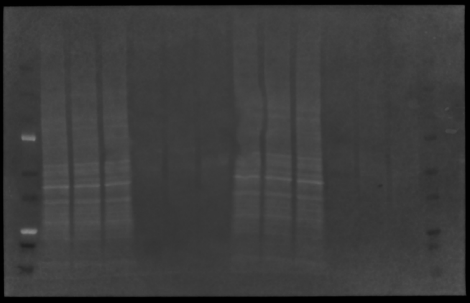

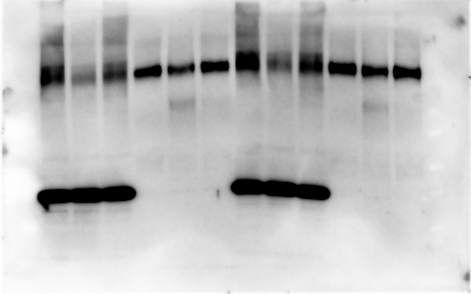
**

*
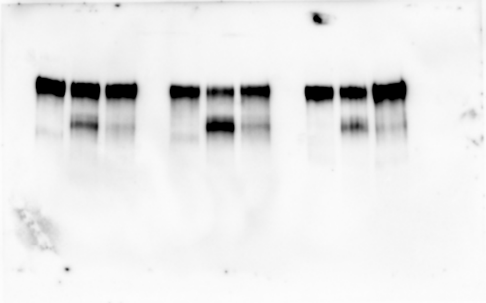

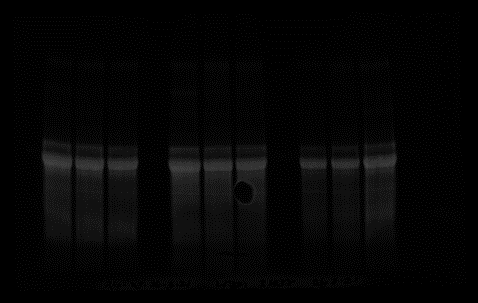
*100-kDa LTBP1 fragment in medium

**Blots from Figure 4A**

Full-length EDA-fibronectin


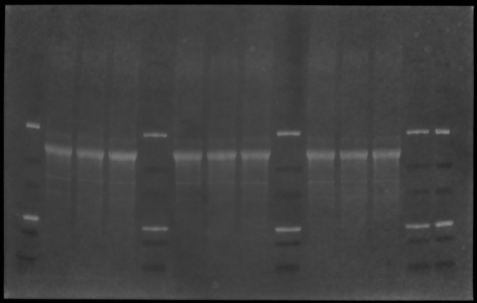

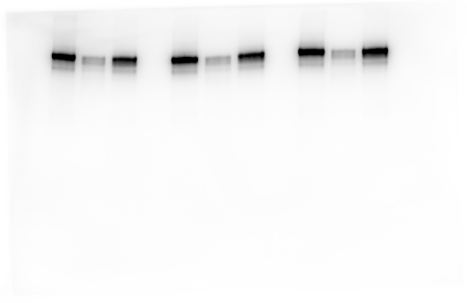


Fibronectin detected by C-terminal antibody


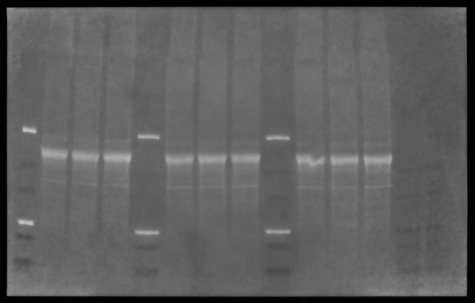

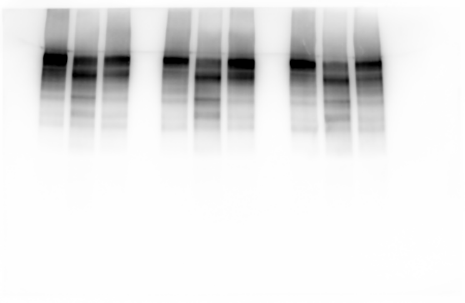


Fibronectin detected by polyclonal antibody


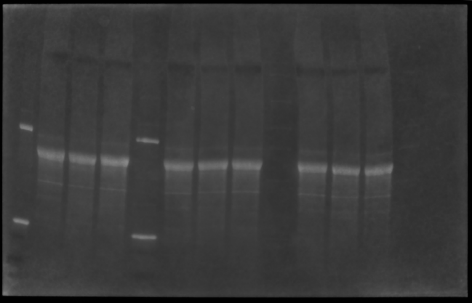

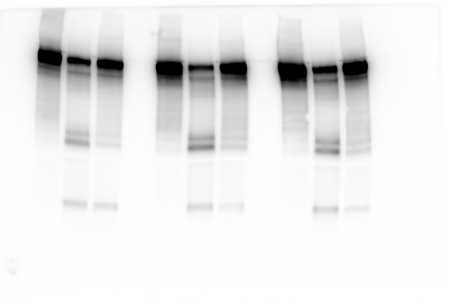


**Blots from Figure 4B**


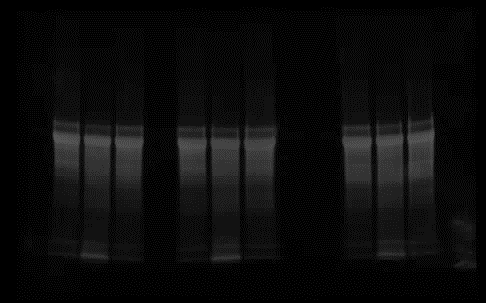

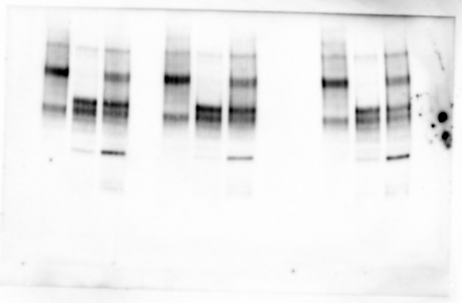
LTBP1

**Blots from Figure 4C and Figure S6**

*Blots are cut horizontally as indicated by a black line. Top blots represent Figure 4C (last six lanes) and Figure S6A (first seven lanes). Bottom blot represents Figure S6B (first seven lanes) and Figure S6C (last six lanes). Representative sections for Figure 4C and Figure S6B are mirrored.*

**
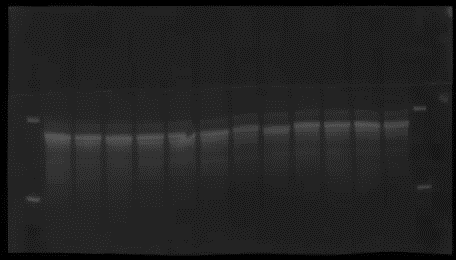

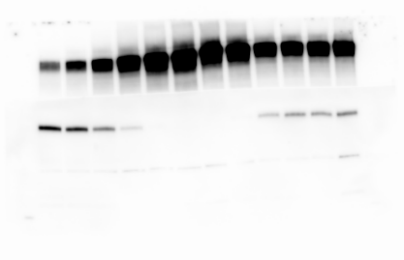
**ADAMTS1

ADAMTS4

**
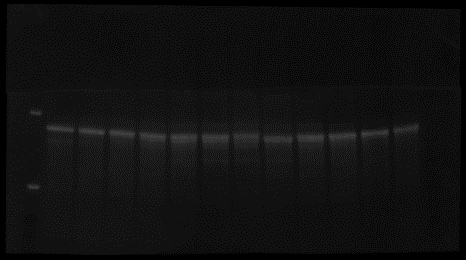
**
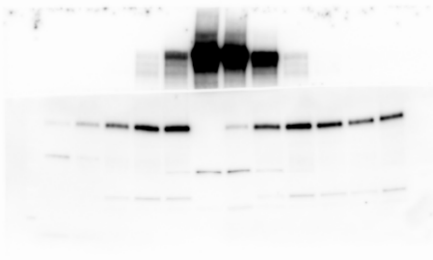


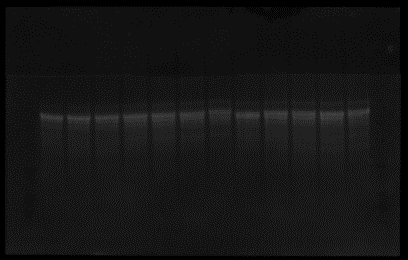

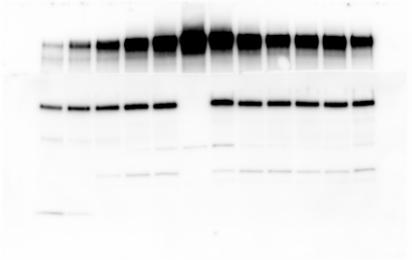
ADAMTS5

**Blots from Figure 5A**
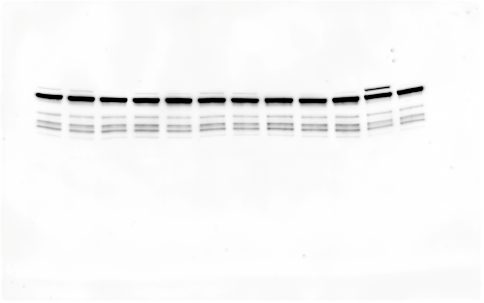


**
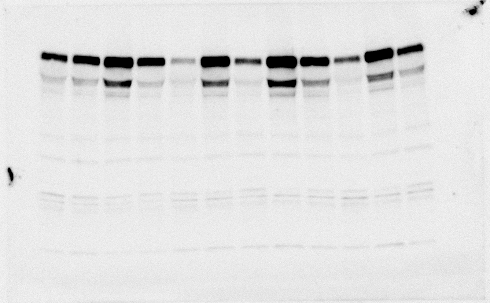
**

**Blots from Figure 5C**

*Black arrows indicate measured band.*

ADAMTS1


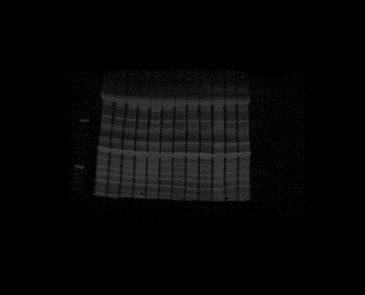
**
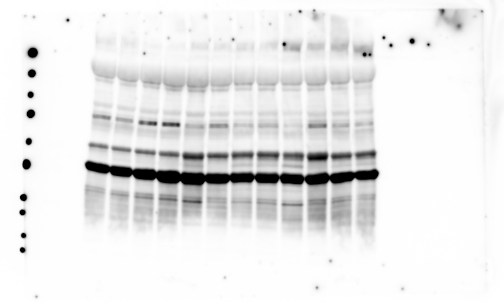
**

→


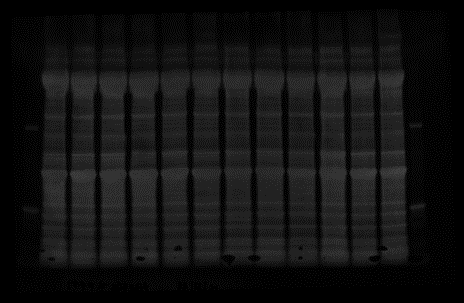
ADAMTS4

→

**
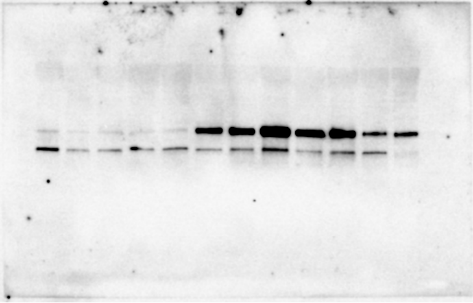
**

ADAMTS5


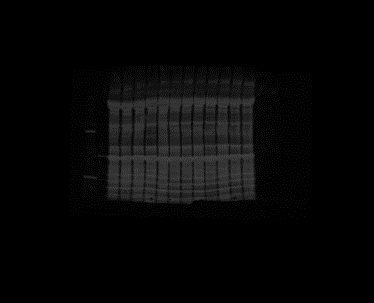

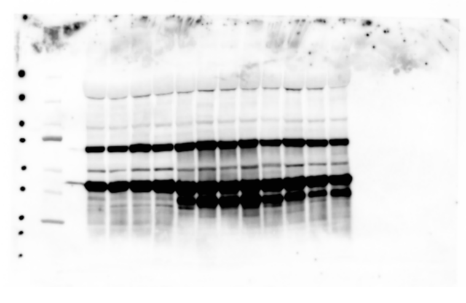


→


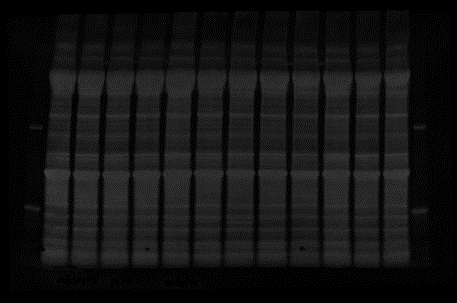

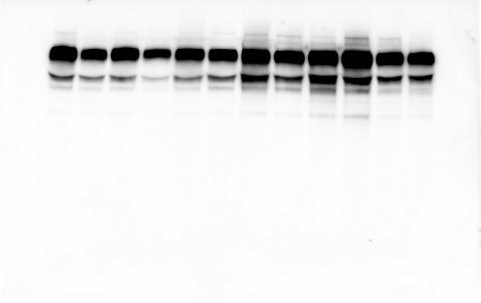
Fibronectin

→


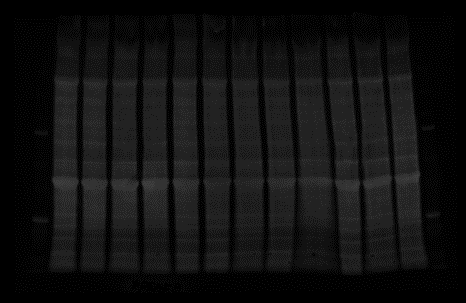
Versican DPEAAE
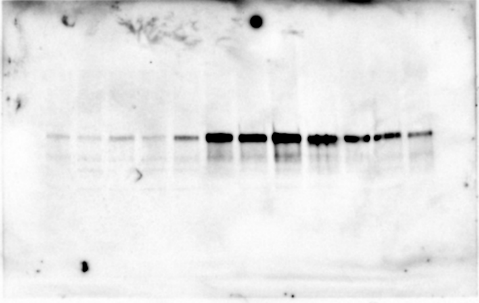


→

**Blots from Figure 5F**

ADAMTS1


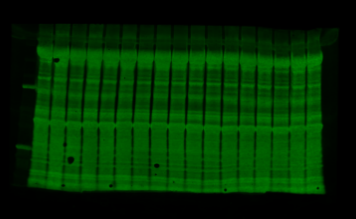
**
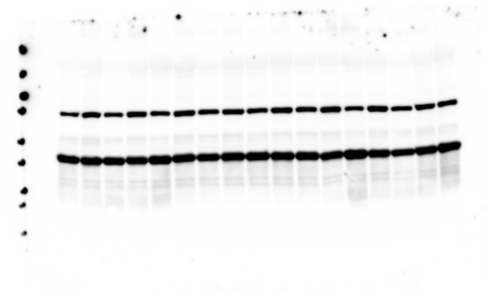
**

ADAMTS4


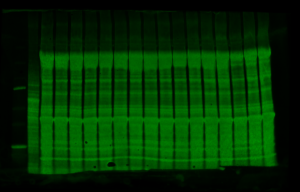
**
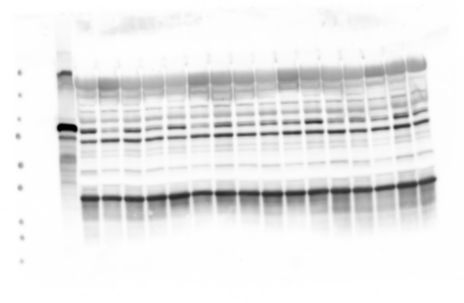
**

ADAMTS5


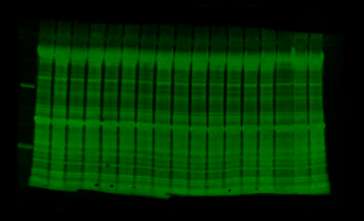
**
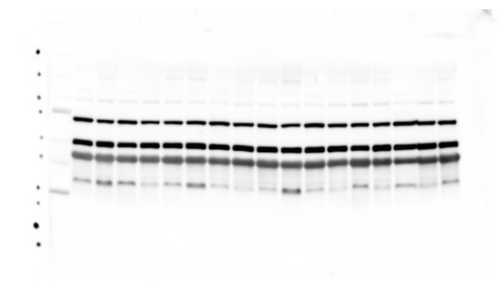
**

**
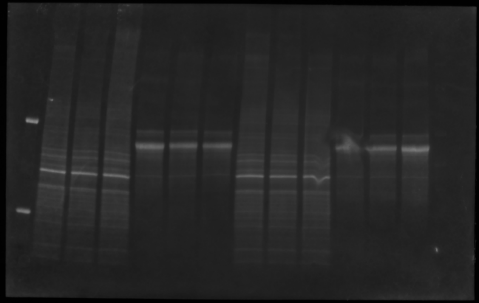
Blots from Figure S7A
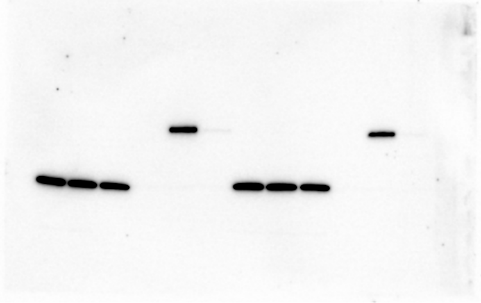
**

**
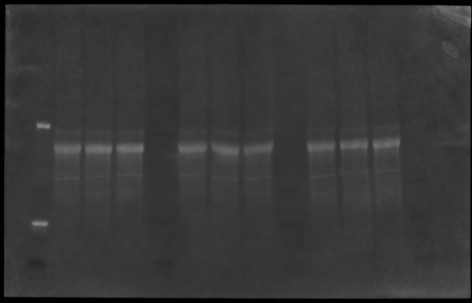
Blots from Figure S7B
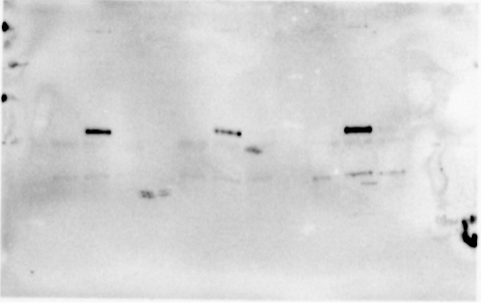
**

**Figure S7C**


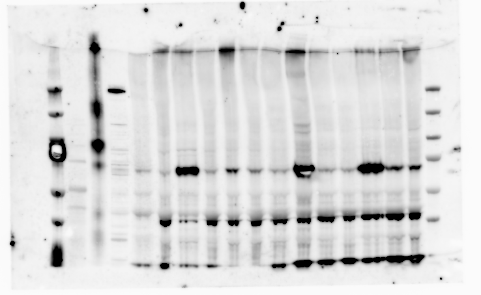


**
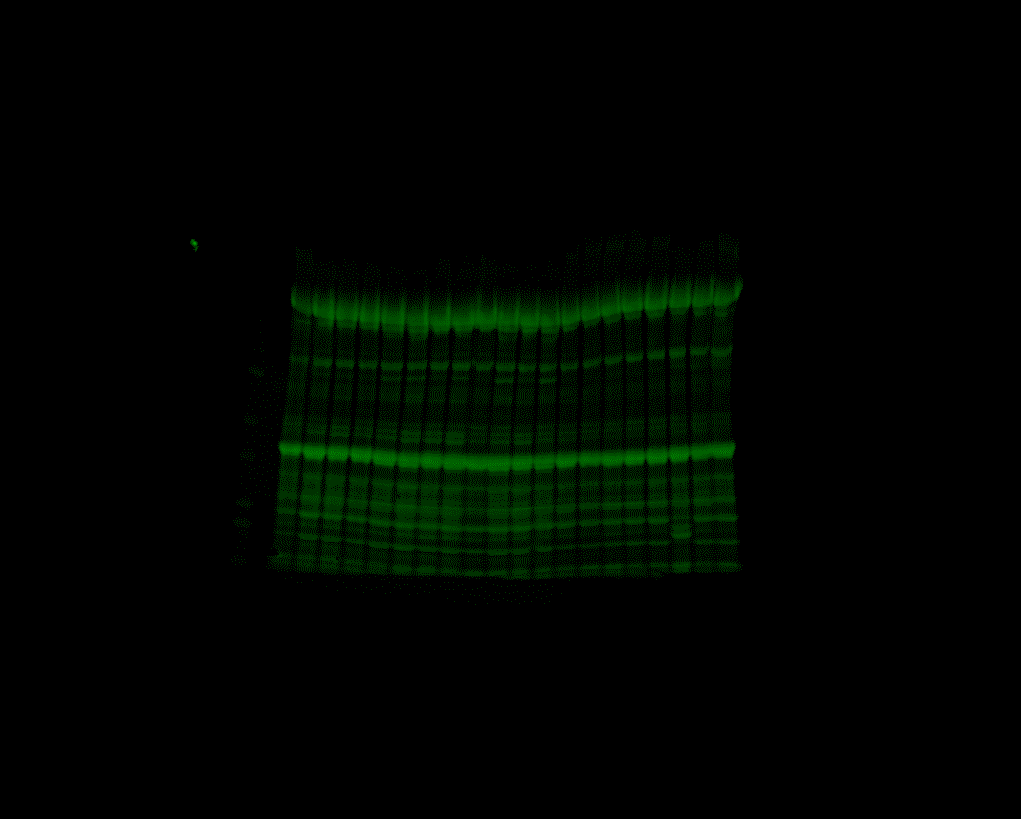
Blots from Figure S7D**
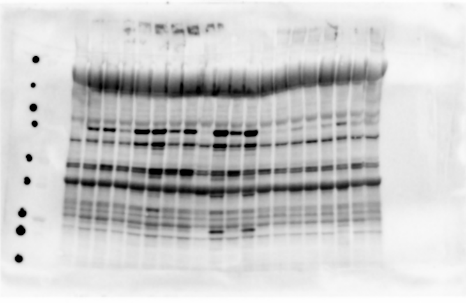


**Blots from Figure S8A**


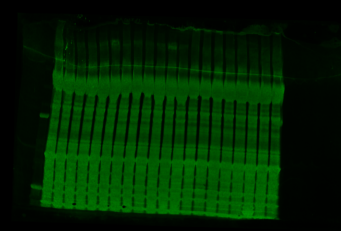
**
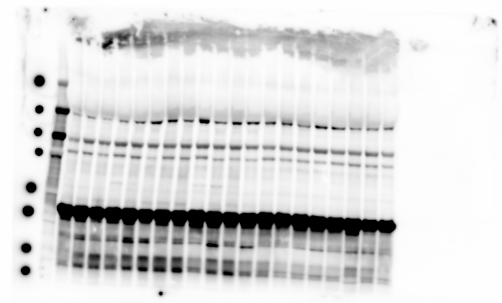
**

**Blots from Figure S8B**


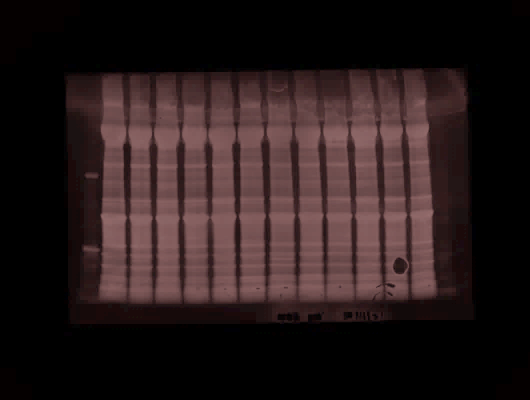

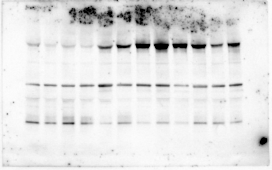


**References**

1. De Savi C, Pape A, Sawyer Y, Milne D, Davies C, Cumming JG, Ting A, Lamont S, Smith PD, Tart J, Page K, Moore P. Orally active achiral N-hydroxyformamide inhibitors of ADAM-TS4 (aggrecanase-1) and ADAM-TS5 (aggrecanase-2) for the treatment of osteoarthritis. *Bioorg Med Chem Lett* 2011;21:3301-3306.

2. Nair AB, Jacob S. A simple practice guide for dose conversion between animals and human. *J Basic Clin Pharm* 2016;7:27-31.

3. Vistnes M, Aronsen JM, Lunde IG, Sjaastad I, Carlson CR, Christensen G. Pentosan polysulfate decreases myocardial expression of the extracellular matrix enzyme ADAMTS4 and improves cardiac function in vivo in rats subjected to pressure overload by aortic banding. *PLoS One* 2014;9:e89621.

4. Andenæs K, Lunde IG, Mohammadzadeh N, Dahl CP, Aronsen JM, Strand ME, Palmero S, Sjaastad I, Christensen G, Engebretsen KVT, Tønnessen T. The extracellular matrix proteoglycan fibromodulin is upregulated in clinical and experimental heart failure and affects cardiac remodeling. *PLoS One* 2018;13:e0201422.

5. Naba A, Clauser KR, Hoersch S, Liu H, Carr SA, Hynes RO. The matrisome: in silico definition and in vivo characterization by proteomics of normal and tumor extracellular matrices. *Mol Cell Proteomics* 2012;11:M111.014647.

6. Didangelos A, Yin X, Mandal K, Saje A, Smith A, Xu Q, Jahangiri M, Mayr M. Extracellular matrix composition and remodeling in human abdominal aortic aneurysms: a proteomics approach. *Mol Cell Proteomics* 2011;10.

7. Patro R, Duggal G, Love MI, Irizarry RA, Kingsford C. Salmon provides fast and bias-aware quantification of transcript expression. *Nat Methods* 2017;14:417-419.

8. Robinson MD, Oshlack A. A scaling normalization method for differential expression analysis of RNA-seq data. *Genome Biol* 2010;11:R25.

9. Yamamoto K, Owen K, Parker AE, Scilabra SD, Dudhia J, Strickland DK. Low density lipoprotein receptor-related protein 1 (LRP1)-mediated endocytic clearance of a disintegrin and metalloproteinase with thrombospondin motifs-4 (ADAMTS-4): functional differences of non-catalytic domains of ADAMTS-4 and ADAMTS-5 in LRP1 binding. *J Biol Chem* 2014;289.

10. McQuin C, Goodman A, Chernyshev V, Kamentsky L, Cimini BA, Karhohs KW, Doan M, Ding L, Rafelski SM, Thirstrup D, Wiegraebe W, Singh S, Becker T, Caicedo JC, Carpenter AE. CellProfiler 3.0: Next-generation image processing for biology. *PLoS Biol* 2018;16:e2005970.

11. Abe M, Harpel JG, Metz CN, Nunes I, Loskutoff DJ, Rifkin DB. An assay for transforming growth factor-beta using cells transfected with a plasminogen activator inhibitor-1 promoter-luciferase construct. *Anal Biochem* 1994;216.

12. Melleby AO, Romaine A, Aronsen JM, Veras I, Zhang L, Sjaastad I, Lunde IG, Christensen G. A novel method for high precision aortic constriction that allows for generation of specific cardiac phenotypes in mice. *Cardiovasc Res* 2018;114:1680-1690.
